# Functional Characterization of Germline Mutations in *PDGFB* and *PDGFRB* in Primary Familial Brain Calcification

**DOI:** 10.1371/journal.pone.0143407

**Published:** 2015-11-23

**Authors:** Michael Vanlandewijck, Thibaud Lebouvier, Maarja Andaloussi Mäe, Khayrun Nahar, Simone Hornemann, David Kenkel, Sara I. Cunha, Johan Lennartsson, Andreas Boss, Carl-Henrik Heldin, Annika Keller, Christer Betsholtz

**Affiliations:** 1 Department of Immunology, Genetics and Pathology, Rudbeck Laboratory, Uppsala University, Dag Hammarskjölds väg 20, Uppsala 75185, Sweden; 2 Institute of Neuropathology, University Hospital Zürich, Zürich University, CH-8091 Zürich, Switzerland; 3 Institute of Diagnostic and Interventional Radiology, University Hospital Zürich, Zürich University, CH-8091 Zürich, Switzerland; 4 Ludwig Institute for Cancer Research, Science for Life Laboratory, Uppsala University, Box 595, SE-75124, Uppsala, Sweden; 5 Division of Neurosurgery, University Hospital Zürich, Zürich University, CH-8091 Zürich, Switzerland; 6 Integrated Cardio Metabolic Centre (ICMC), Karolinska Institute, Novum, SE-141 57 Huddinge, Stockholm, Sweden; University of Michigan School of Medicine, UNITED STATES

## Abstract

Primary Familial Brain Calcification (PFBC), a neurodegenerative disease characterized by progressive pericapillary calcifications, has recently been linked to heterozygous mutations in *PDGFB* and *PDGFRB* genes. Here, we functionally analyzed several of these mutations *in vitro*. All six analyzed *PDGFB* mutations led to complete loss of PDGF-B function either through abolished protein synthesis or through defective binding and/or stimulation of PDGF-Rβ. The three analyzed *PDGFRB* mutations had more diverse consequences. Whereas PDGF-Rβ autophosphorylation was almost totally abolished in the *PDGFRB* L658P mutation, the two sporadic *PDGFRB* mutations R987W and E1071V caused reductions in protein levels and specific changes in the intensity and kinetics of PLCγ activation, respectively. Since at least some of the *PDGFB* mutations were predicted to act through haploinsufficiency, we explored the consequences of reduced *Pdgfb* or *Pdgfrb* transcript and protein levels in mice. Heterozygous *Pdgfb* or *Pdgfrb* knockouts, as well as double *Pdgfb* ^+/-^;*Pdgfrb* ^+/-^ mice did not develop brain calcification, nor did *Pdgfrb* ^*redeye/redeye*^ mice, which show a 90% reduction of PDGFRβ protein levels. In contrast, *Pdgfb* ^*ret/ret*^ mice, which have altered tissue distribution of PDGF-B protein due to loss of a proteoglycan binding motif, developed brain calcifications. We also determined pericyte coverage in calcification-prone and non-calcification-prone brain regions in *Pdgfb* ^*ret/ret*^ mice. Surprisingly and contrary to our hypothesis, we found that the calcification-prone brain regions in *Pdgfb* ^*ret/ret*^ mice model had a higher pericyte coverage and a more intact blood-brain barrier (BBB) compared to non-calcification-prone brain regions. While our findings provide clear evidence that loss-of-function mutations in *PDGFB* or *PDGFRB* cause PFBC, they also demonstrate species differences in the threshold levels of PDGF-B/PDGF-Rβ signaling that protect against small-vessel calcification in the brain. They further implicate region-specific susceptibility factor(s) in PFBC pathogenesis that are distinct from pericyte and BBB deficiency.

## Introduction

The role of the platelet-derived growth factors (PDGFs) and their tyrosine kinase receptors (PDGFRs) has been extensively studied in the developing organism [1,2]. Of the two prototypical PDGFRs, PDGF-Rα and PDGF-Rβ, the latter has been especially implicated in blood vessel formation and early hematopoiesis. PDGF-Rβ signaling, initiated by the secretion of PDGF-B ligand by endothelial cells, is paramount for the recruitment of PDGF-Rβ positive vascular smooth muscle cells and pericytes during angiogenesis [3,4]. In mice, homozygous knockouts of either receptor or ligand display widespread vascular alterations leading to perinatal death [1,5,6]. Heterozygous knockouts, on the contrary, bear no or subtle phenotypic changes [7,8].

In comparison, the pathogenic roles of PDGFs are still poorly understood. Regarding PDGF-B, increased PDGF-Rβ signaling has been demonstrated in diseases like cancer, vascular inflammation, and tissue fibrosis [1]. Focal uncontrolled PDGF-Rβ signaling due to somatic genetic aberrations has severe consequences in humans, playing a causative role in diseases such as *dermatofibrosarcoma protuberans* [9], gastric cancer and leukemia [10]. Recently, infantile myofibromatosis (IMF), a disease characterized by proliferative fibrous tumors during childhood, was linked to putative gain-of-function germ-line mutations in *PDGFRB* [11–13]. The simultaneous discovery that mutations in *PDGFB* and *PDGFRB* can also cause primary familial brain calcification (PFBC) demonstrates new functions of PDGF-Rβ signaling that could not have been predicted based on existing knowledge about PDGF-B/ PDGF-Rβ functions, demonstrating that there is still much to be learnt about PDGF biology [14,15].

**Fig 1:**
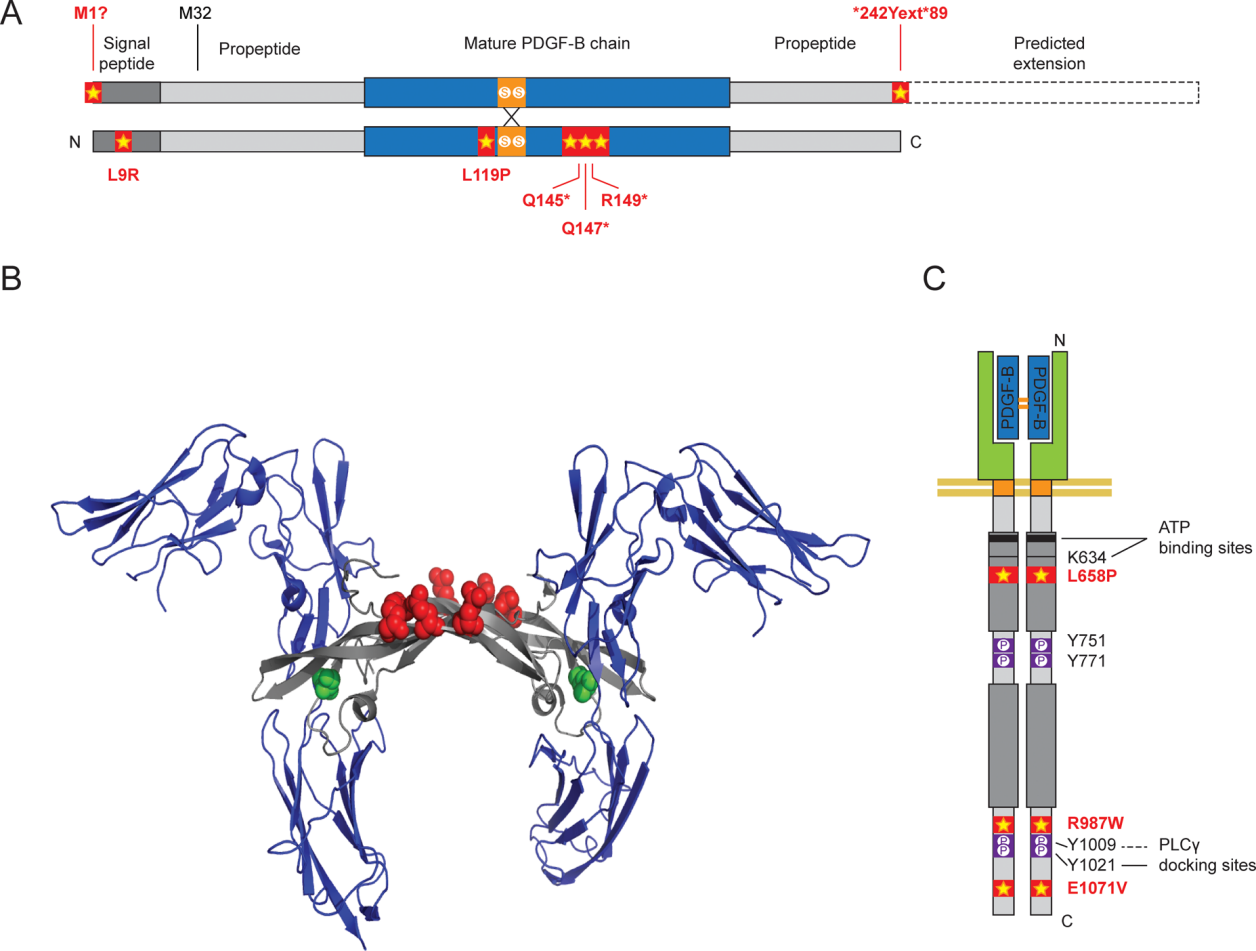
Overview of PFBC-related *PDGFB* and *PDGFRB* mutations. (A) In-scale schematic representation of a PDGF-BB dimer with the location of the known PFBC-associated mutations. Cysteine residues involved in interchain disulfide bonds are indicated in orange. The relative position of different mutations is indicated by stars. The predicted protein extension due to the stop codon mutation is indicated by dashed boxes. (B) Location of the main point mutations in *PDGFB* in the PDGF-BB:PDGF-Rβ complex. Ribbon diagram of two PDGF-Rβ proteins (in blue) in complex with dimeric PDGF-B (in grey). The location of the 3 stop mutations Q145*, Q147* and R149* are indicated in red. The location of the L119P missense mutation is indicated in green. The image was created from PDB 3MJG (Platelet-derived growth factor subunit B) using PyMOL (The PyMOL Molecular Graphics System, Version 1.2r3pre, Schrödinger, LLC. http://pymol.sourceforge.net/faq.html). (C) Schematic representation of a PDGFRβ dimer with the location of the known PFBC-associated mutations. Ig-like C2-type domains are indicated by oval shapes. The split tyrosine-kinase domain is indicated by dark grey boxes. Tyrosine autophosphorylation sites Y751, Y771, 71009 and Y1021 assessed in the article are indicated in purple. ATP-binding sites are indicated in black, including the K634 residue mutated in our kinase-dead negative control. The relative position of the mutations is indicated by stars.

PFBC, previously referred to as Fahr’s disease or idiopathic basal ganglia calcification (IBGC), is a neurological disease characterized by age-dependent perivascular calcifications in the brain and clinically heterogeneous symptoms ranging from Parkinsonism to cognitive impairment [16]. This rare disorder has an autosomal-dominant pattern of inheritance [14,17]. The first PFGC mutation in either *PDGFB* or *PDGFRB* to be described was the L658P (c.1973T>C) missense mutation in the protein kinase catalytic domain of PDGF-Rβ, which segregated with the disease in a large family with 13 PFBC patients [14]. Following this finding, two additional and potentially pathogenic mutations in the C-terminal domain of *PDGFRB*, R987W (c.2959C>T), and E1071V (c.3212A>T), were found in sporadic cases [14,17]. Lately, a R695C (c.2083C>T) mutation was described in a single patient from an autopsy series of PFBC [18]. Further strengthening the link between PFBC and PDGF-B/ PDGF-Rβ signaling, six different nonsense and missense mutations in the gene encoding PDGF-B were identified in six PFBC families of different ancestry [15]. A seventh nonsense mutation was reported in a sporadic PFBC case [19]. These mutations include two missense mutations in the initiation and in the stop codons, M1? (c.3G>A) and *242Yext*89 (c.726G>C), the latter resulting in a 89 amino acid extension; one L9R (c.26T>G) missense mutation in the signal peptide; and one missense and three nonsense mutations in the sequence coding for the growth factor domain (L119P (c.356T>C); Q145* (c.433C>T); Q147* (c.439C>T) and R149* (c.445C>T), respectively) [15,19]. Recently, a heterozygous intragenic deletion in *PDGFB* involving exons 2–5 of *PDGFB* was described in a sporadic PFBC patient [20]. Fig 1 provides an overview of all *PDGFB* and *PDGFRB* mutations.

The predicted deleterious consequences of the nonsense mutations identified in *PDGFB* and the dual involvement of *PDGFB* and *PDGFRB* led to the assumption that all PFBC mutations were loss-of-function [15]. Two recent reports showed that some of the *PDGFRB* mutations described in PFBC indeed cause defective receptor autophosphorylation, protein stability and/or downstream signaling [18,21]. However, consequences of the *PDGFB* mutations found in PFBC cases have previously not been analyzed. Interestingly, mice carrying hypomorphic *Pdgfb* alleles where PDGF-B retention motif has been deleted (*Pdgfb* ^*ret/ret*^ mice) develop brain calcifications that show age-related expansion [15].

While there is a link between impairment of PDGF-B signaling and familial PFBC, it is unclear what kind and extent of impairment that is required to trigger PFBC. Since defective PDGFB signaling also leads to severely reduced pericyte counts and a dysfunctional blood-brain barrier in the adult mouse brain, we previously hypothesized a causal relationship between these defects and the observed brain calcifications. However, it remains possible that the pericyte/BBB deficiencies and brain calcifications represent independent consequences of impaired PDGF-B signaling. Whereas all human *PDGFB* mutations described to-date are dominant, and at least some of them able to act through haploinsufficiency, the *Pdgfb* ^*ret/ret*^ mouse model of PFBC is clearly acting differently. We therefore characterized human *PDGFB* and *PDGFRB* mutations *in vitro*. Using biochemical and cellular approaches, we analyzed the effects of the point mutations in *PDGFB* on expression and secretion of PDGF-B, activation of PDGF-Rβ, and actin reorganization in primary human pericytes. We confirmed and complemented previous findings on the effects of L658P, R987W and E1071V mutations in *PDGFRB* by assessing receptor tyrosine kinase autophosphorylation, activation of downstream signal transduction cascades and cell migration. Moreover, we analyzed the consequences of different degrees of *Pdgfb* and/or *Pdgfrb* genetic insufficiency on pericyte coverage and blood-brain barrier integrity in mice.

## Results

### *PDGFB* mutations are null or loss-of-function mutations

To study the functional consequences of *PDGFB* mutations described in PFBC (Fig 1A and 1B), cDNA sequences carrying the different mutations identified in six PFBC families were introduced into the pcDNA3.1 vector, transfected into human embryonic kidney (HEK) 293 cells, and after G-418 selection, stably expressing clones were generated. Wild-type *PDGFB* cDNA and empty pcDNA3.1 vector served as positive and negative controls, respectively. In order to assess the expression and secretion of mutant PDGF-B, transfected cells were lysed for RNA or protein extraction. In separated experiments, serum-free conditioned medium was collected and analyzed.

**Fig 2:**
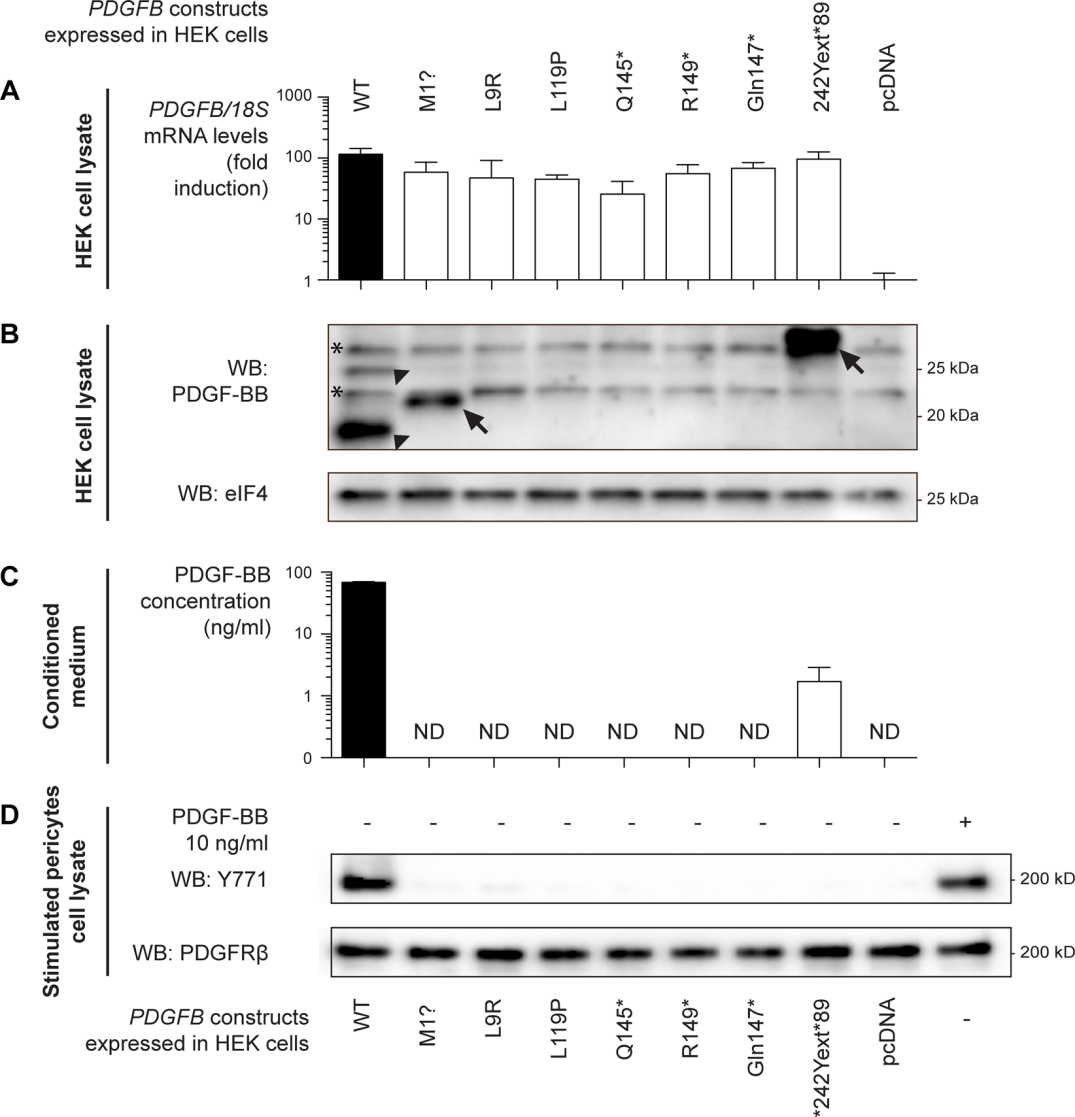
PFBC *PDGFB* mutations result in loss of detectable and/or functional PDGF-BB. (A) qPCR analysis for human PDGFB in stably transfected HEK293 cells. Individual vectors expressing each of the mutations identified in PFBC families were stably transfected into HEK cells. Wild-type (WT) *PDGFB*-expressing construct and empty vector (pcDNA) were used as positive and negative controls, respectively. mRNA levels are shown as fold increase over basal expression of PDGF-B in HEK cells. 18s Ribosomal RNA was used for normalization. Error bars indicate standard deviation of three independent experiments. (B) Detection of PDGF-B protein expression in stably transfected HEK293 cells. Immunoblot of HEK293 cell lysates showing expression of the different *PDGFB* constructs. The arrowheads indicate the 2 bands corresponding to PDGF-B in the wild-type condition, probably representing fully and partially proteolytically processed PDGF-B chains. Arrows indicate specific bands migrating slower due to a mutation-induced size shift. Non-specific bands in the background are marked with asterisks. (C) ELISA assay for detection of PDGF-BB in conditioned medium of stably transfected HEK cells. HEK cells overexpressing the different PFBC mutations were serum-starved for 24 hours prior to collection of their growth medium. An ELISA specific for human PDGF-BB was performed to detect secreted PDGF-BB. Error bars indicate the standard deviation over three individual measurements. *ND*: Not detected. (D). Western blot of autophosphorylation of PDGF-Rβ in human brain pericytes (HBP). HBPs were serum-starved overnight, cooled on ice and their medium replaced by either cooled conditioned medium from B-C, or cooled pericyte medium supplemented with 10 ng/ml PDGF-BB (right lane). After 1 hour-incubation on ice, cells were lysed and immunoblots were performed on duplicate membranes using anti-phospho-PDGF-Rβ (Y771) and anti-total PDGF-Rβ (28E1) antibodies.

All *PDGFB* constructs produced comparable levels of *PDGFB* mRNA in HEK293 cells (Fig 2A). To assess protein expression, a polyclonal anti-PGDF-B antibody was used in Western blotting of cell lysates. Wild-type PDGF-B was detected as a major ≈14-kDa and a minor 25-kDa species probably representing fully and partially proteolytically processed PDGF-B chains, respectively (Fig 2B, arrowheads) [22]. Among the expression vectors expressing mutated PDGF-B, PDGF-B antibodies detected specific PDGF-B products only from the initiation and stop codon mutants, respectively (M1? and *242Yext*89). The ≈21–22 kDa PDGF-B protein in M1?-transfected cells (Fig 2B, arrow in second lane) might be explained by an alternative translational start site. Of the several in-frame ATG codons that occur up- and downstream of the native ATG, the M32 in the PDGF-B precursor would generate a 158 amino acid-long product with a MW of ≈21.5 kDa (if no further N-terminal or C-terminal processing occurs). The ≈30 kDa-protein generated by the *242Yext*89 mutation (Fig 2B, arrow in eighth lane), has a size that would be predicted from an 89 residue-long C-terminal extension of a precursor protein in which N-terminal processing occurred normally (Figs 2B and 1A). Since the PDGF-B precursor protein is known to be differentially processed depending on whether it is routed for secretion or degradation [22], we did not address the structure of the intracellular mutant PDGF-B proteins further, but instead focused on the presence of a secreted biologically active PDGF-B protein. None of the other constructs produced any detectable PDGF-B protein (Fig 2B).

**Fig 3:**
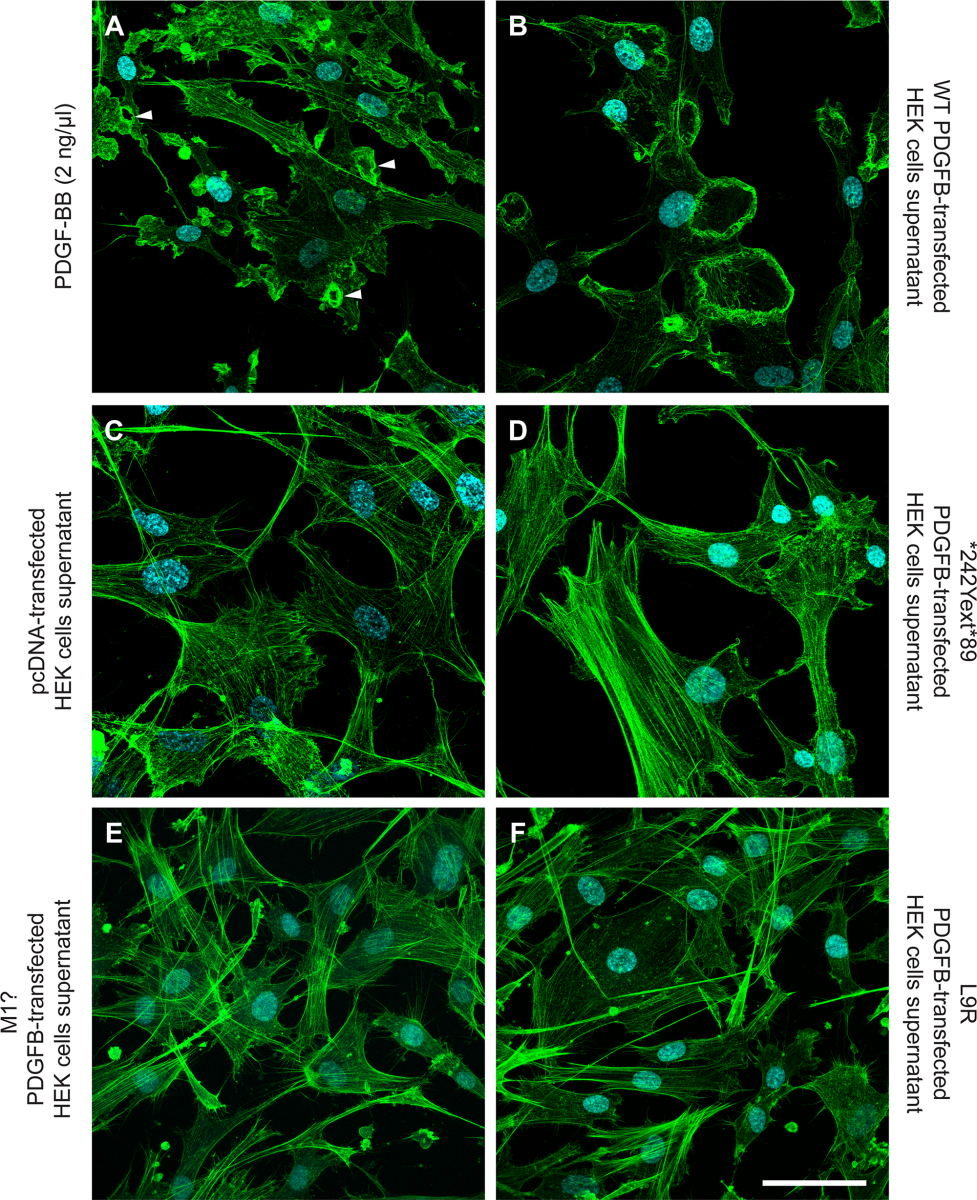
Conditioned medium from mutant *PDGFB*-transfected HEK cells fails to induce membrane ruffles in human brain pericytes. After overnight serum starvation, human brain pericytes (HBP) were cooled on ice and exposed to cooled conditioned medium from HEK cells (described in Fig 1B and 1C) for 15 minutes, then warmed up in at 37°C for 30 min and fixed for phalloidin staining. A low concentration of exogenous PDGF-BB (2 ng/ml) in serum-free pericyte medium (A) and conditioned medium from wild-type *PDGFB-*transfected HEKs (B) were used as a positive controls. Supernatant from pcDNA-transfected HEKs was used as a negative control (C). The first two conditions induced widespread circular ruffles (arrowheads), which were absent in the negative control. Likewise, HBP treated with conditioned medium from mutant *PDGFB* transfected HEKs did not show any ruffles: (D) *242Yext*89 mutation, (E) M1? mutation and (F) L9R mutation. Cyan: DAPI. Green: Alexa 488 conjugated phalloidin. Scale bar: 30 μm.

Using a PDGF-Rβ-binding based ELISA to measure PDGF-BB concentration in the conditioned media, we found that only the stop codon mutation (*242Yext*89) yielded detectable amounts of secreted PDGF-BB (2.05 ± 0.64 ng/ml), however at 50-fold lower levels than in wild-type *PDGFB*-transfected HEKs (Fig 2C). To assess the ability of the stop codon mutant (*242Yext*89) to activate PDGF-Rβ, and to rule out the presence of functional, yet undetected, PDGF-BB in the supernatant from mutant-transfected HEKs, serum-starved human brain pericytes (HBPs) were exposed to conditioned medium collected from HEK cells expressing different PDGFB mutants. While recombinant PDGF-BB and conditioned medium from wild-type *PDGFB*-transfected HEKs induced detectable PDGF-Rβ phosphorylation (Fig 2D first and last lane, respectively), media from all PDGFB mutant-transfected, as well as the empty vector-transfected, HEKs failed to induce tyrosine phosphorylation of PDGFRβ above background levels (Fig 2D lanes 2–8 and 9, respectively). Likewise, conditioned media from HEK cells expressing the PFBC *PDGFB* mutations failed to induce rearrangement of the actin cytoskeleton (Fig 3D and S1 Fig). In marked contrast, conditioned medium from wild-type *PDGFB*-transfected HEKs, or PDGF-BB at concentrations as low as 2 ng/ml, induced a profound reorganization of the actin cytoskeleton in HBPs (Fig 3A and 3B). Actin remodeling anifested as a loss of stress fibers and the formation of peripheral and circular ruffles, the latter being one of the earliest, most sensitive and specific cellular events following PDGF-BB stimulation [23–25].

In conclusion, all six analyzed *PDGFB* mutations lead to a complete abolishment of a functional PDGF-B protein when expressed *in vitro* individually and in the absence of wild-type PDGF-B. Additionally, most of the mutations appeared to cause a complete loss of PDGF-B protein production (i.e. *null* mutations), suggesting that *PDGFB* haploinsufficiency could be a sufficient cause of PFBC (overview in S1 Table).

### The L658P mutation in *PDGFRB* abolishes PDGF-BB dependent receptor autophosphorylation

To study the functional consequences of *PDGFRB* mutations, pcDNA3.1 constructs containing 3 different *PDGFRB* mutations identified in PFBC patients (L658P, R987W and E1071V, Fig 1C) were transfected into porcine aortic endothelial (PAE) cells. Additional transfections of constructs encoding wild-type PDGF-Rβ, kinase-dead (KD) PDGF-Rβ (K634A) [26] and empty expression vector served as positive and negative controls.

**Fig 4:**
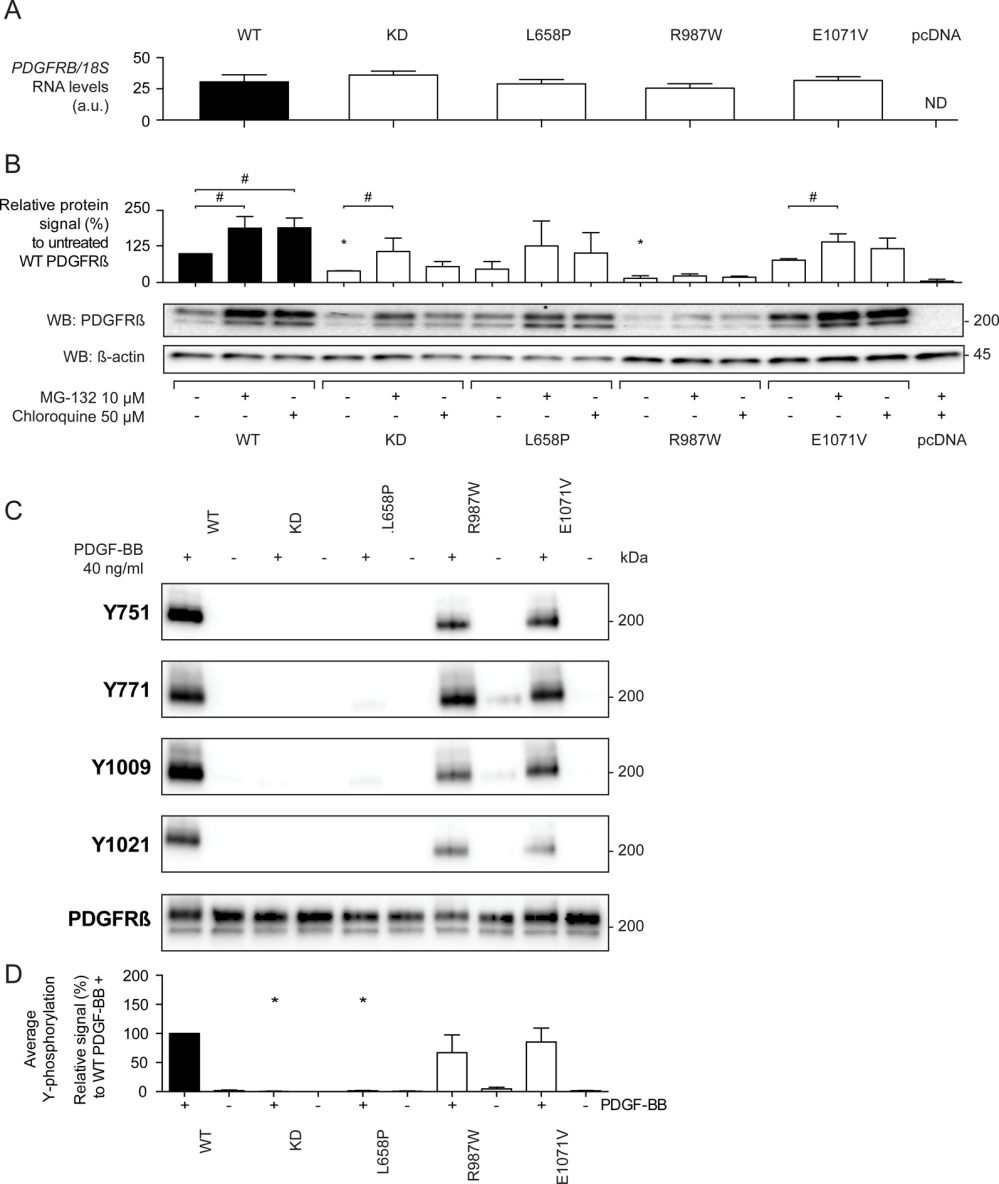
Expression and autophosphorylation of PDGF-Rβ mutants in PAE cells. PAE cells were stably transfected with vectors expressing the different PFBC mutations found in *PDGFRB*. (A) After mRNA extraction, the total expression of human *PDGFRB* was detected with qPCR. Error bars indicate standard deviation between 3 independent experiments. ND: Not detected. (B) Immunoblot demonstrating the protein expression levels of the different *PDGFRB* mutants. Stable clones of mutant *PDGFRB* expressing PAE cells were treated with a proteasomal inhibitor (MG-132), lysosomal inhibitor (chloroquine) or vehicle for 4 hours before lysis. Total levels of PDGF-Rβ were visualized with anti-total PDGF-Rβ (28E1) antibody. The graphs indicate relative expression level of the inhibitor-treated conditions *vs* control condition for three individual experiments. Error bars indicate standard deviation. *p<0.05 compared to basal wild type (WT) expression, ^#^ *p*<0.05 when comparing inhibitor-treated *vs* control condition for each mutant. (CD) Autophosphorylation of the PDGF-Rβ mutants. PAE cells stably expressing mutant PDGF-Rβ were exposed to 40 ng/ml of exogenous PDGF-BB for 60 minutes after cooling on ice. A wild-type *PDGFRB*-expressing construct was used as a positive control, while a kinase dead (KD) variant was used as a negative control. Cell lysates were adjusted to yield a comparable amount of PDGF-Rβ signal. (C) Representative western blot demonstrating autophosphorylation of the different mutants on four residues. (D) Quantification of PDGF-Rβ autophosphorylation signal from tyrosine residues 751, 771, 1009 and 1021 using phospho-specific antibodies. Signals were normalized over total levels of PDGF-Rβ protein, and expressed as a percentage of wild-type PDGF-Rβ autophosphorylation. The graph represents the averaged results from the 4 tyrosine residues that were assessed in three independent experiments. *p<0,05 when compared to the positive control (WT PDGF-Rβ).

Stable transfection of *PDGFRB* constructs into PAE cells resulted in *PDGFRB* mRNA production, while *PDGFRB* mRNA was undetectable in the empty vector control (Fig 4A). There was no significant difference in *PDGFRB* mRNA levels between the different constructs, indicating similar transfection and transcription efficiencies between expression vectors encoding mutant or wild-type *PDGFRB*. The relative PDGF-Rβ protein expression level was assessed by Western blotting. The KD and R987W PDGF-Rβ mutants yielded a significantly weaker signal (40 and 15%, respectively) as compared with wild-type PDGF-Rβ, whereas the expression level of L658P and E1071V PDGF-Rβ mutants did not differ from wild-type PDGF-Rβ. Proteasome and lysosome inhibitors (MG-132 and chloroquine, respectively) did not significantly increase the amount of PDGF-Rβ R987W protein, suggesting that the lower protein level was not due to increased turnover of the protein (Fig 4B).

Upon ligand binding, PDGF-Rβ dimerizes and autophosphorylates on as many as 13 cytoplasmic tyrosine residues [1,27]. We assessed four of these (Y751, Y771, Y1009 and Y1021). PDGF-BB treatment of wild-type *PDGFRB*-transfected cells induced a strong phosphorylation of PDGF-Rβ in all four tested tyrosine residues. Conversely, there was no detectable phosphorylation of KD PDGF-Rβ, despite a total PDGF-Rβ protein signal was equivalent to the wild-type condition (Fig 4C and 4D and S2 Fig). Assessment of PFBC PDGF-Rβ mutants showed that similar to the KD condition, the L658P mutant displayed a nearly complete abolition of the phospho-PDGF-Rβ signal following PDGF-BB treatment in all tested tyrosine residues. In contrast, the R987W and E1071V mutants behaved like wild-type PDGF-Rβ with regard to phosphorylated/total protein ratio in the PDGF-BB-treated condition (Fig 4C and 4D). Detailed analysis of each tyrosine residue yielded the same results, with the exception of slightly weaker Y1009 and Y1021 phosphotyrosine signals in the R987W mutant (S2 Fig).

### Downstream signaling is compromised by *PDGFRB* L658P mutation and, to a lesser extent, by R987W and E1071V mutation

**Fig 5:**
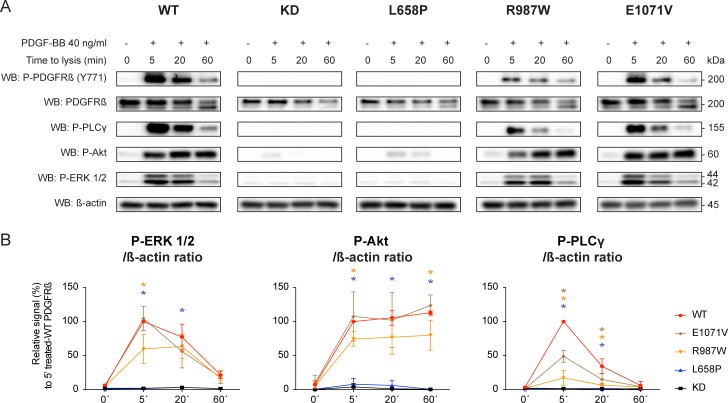
Effect of *PDGFRB* mutations on PDGF-B signaling. (A) Western blot for known downstream targets of PDGF-B signaling. PAE cells were stimulated with 40 ng/ml PDGF-BB for the indicated time periods. Autophosphorylation of PDGF-Rβ mutants was investigated with a phosphospecific antibody raised against tyrosine 771, while downstream PDGF-BB signaling was assessed with antibodies directed against phosphorylated activated forms of ERK 1/2, Akt and PLCγ. β-actin was used as a loading control. (B). To quantify ERK 1/2, Akt and PLCγ activations over time, the signals were normalized over β-actin levels and plotted against time. Error bars indicate the standard deviation of three independent experiments. *p˂0,05 when compared to wild-type PDGFRβ (red), with the color of the star indicating the mutant PDGFRβ that is being compared to wild-type PDGFRβ (blue: L658P, yellow: R987W, brown: E1071V).

Within minutes of PDGF-Rβ autophosphorylation, many intracellular signaling pathways are engaged. These include Ras-MAPK, PI3K/Akt and PLCγ [1,27,28]. As expected, phosphorylation of ERK1/2 and Akt was reduced or absent in the cells expressing the L658P mutant after PDGF-BB stimulation (Fig 5A). The R987W PDGF-Rβ mutation showed lower ERK1/2 and Akt activation at 5 minutes after ligand stimulation. More strikingly, PLCγ activation was markedly decreased at all time points in cells expressing the R987W and E1071V mutants expressing cells after PDGF-BB exposure as compared to wild-type PDGF-Rβ (Fig 5A and 5B).

**Fig 6:**
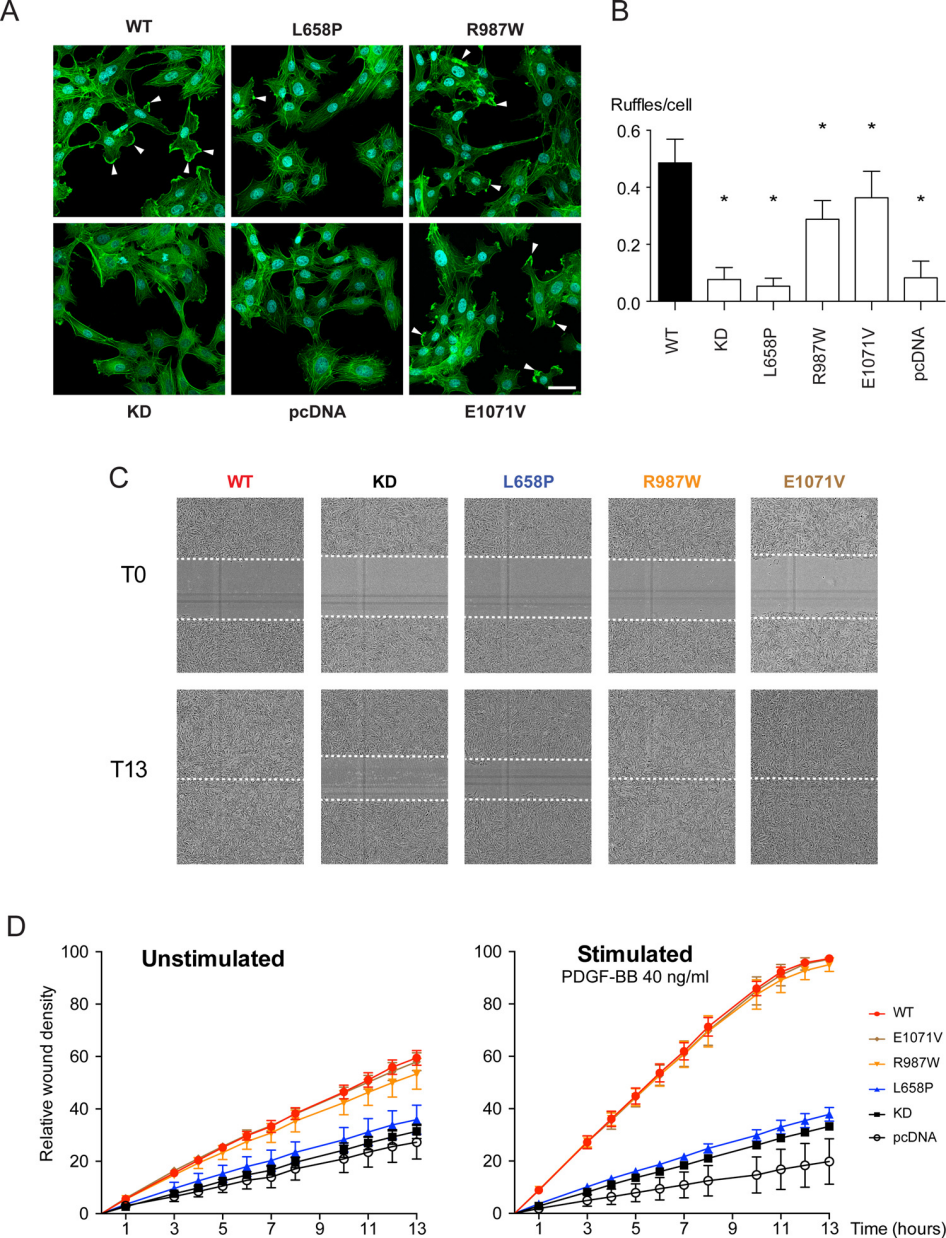
Effect of *PDGFRB* mutations on membrane ruffle formation and wound healing. (AB) PDGF-BB—induced membrane ruffling of stably transfected PAE cells expressing mutant PDGF-Rβ receptor. (A) Fluorescent labeling of the actin cytoskeleton in PAE cells after 30 minutes of PDGF-BB exposure. Arrowheads indicate peripheral membrane ruffles. Cyan: DAPI, green: Alexa 488-conjugated phalloidin. Error bar: 30 μm. (B) The total amount of ruffles was counted in 20 fields per condition, and normalized over the total amount of cells. *p˂0.05 as compared to wild-type *PDGFRB*-expressing PAE cells. Empty vector (pcDNA) and KD *PDGFRB*-transfected cells were used as negative controls. (CD) Wound healing assays of stably transfected PAE cells expressing different mutant PDGFRβ receptors. Confluent monolayers of PAE cells expressing different *PDGFRB* constructs were scratched using the WoundMaker™ and wound closure was monitored automatically every hour for 13 hours with the IncuCyte Zoom®. (C) Representative images of the wound at 0 and 13 hours of PDGF-BB stimulation. (D) Quantification of the increase in relative wound density within 13 hours. Error bars indicate standard deviation between 6 individual scratches, 2 images per scratch.

To elucidate the influence of the PDGF-Rβ mutations on PAE cell behavior, we investigated the ability of these cells to form membrane ruffles when treated with PDGF-BB. Although PAE cells expressing wild-type, or R987W or E1071V-mutated PDGF-Rβ all displayed membrane ruffles after PDGF-BB stimulation, the number of ruffles were slightly but significantly lower in cells expressing R987W or E1071V mutants. In contrast, membrane ruffles were rare in PDGF-BB treated PAE cells expressing the L658P mutant or in empty vector transfected control cells (Fig 6A and 6B).

Since membrane protrusions are known to play a role in cell migration, we investigated if the compromised inability of the PAE cells to form membrane ruffles correlated with any impairment in cell migratory potential. Whereas wild-type *PDGFRB*-transfected cells grown to confluency did close a ~730 μm-wide scratch wound to completion (100%) in 13 hours upon serum starvation followed by PDGF-BB stimulation, the relative wound closure was less than 40% for empty vector and KD *PDGFRB*-transfected cells (Fig 6D) with no significant effect over background of PDGF-BB stimulation (Fig 6C). Cells transfected with vector encoding PDGF-Rβ L658P behaved similar to cells transfected with empty vector or vector encoding KD PDGF-Rβ. Interestingly, cells expressing R987W and E1071V PDGF-Rβ mutants did not show differences from cells expressing wild-type PDGF-Rβ in scratch wound assay (Fig 6C and 6D and S1 Movie).

Overall, of all the analyzed *PDGFRB* mutations, only the L658P mutation behaved as a complete loss-of-function mutation. R987W and E1071V mutants retained significant biological activity in different cell assays. However, R987W mutation caused lower protein synthesis and/or decreased stability of the receptor, whereas the E1071V mutant exerted a limited negative effect on PDGFRβ downstream signaling (overview in S1 Table).

### Genetic haploinsufficiency for *Pdgfb* and/or *Pdgfrb* or a substantial decrease in PDGF-Rβ protein do not trigger brain calcification in mice

Whereas most of the *PDGFB* mutations in PFBC lead to complete loss of PDGF-B protein synthesizing capacity from the mutant *PDGFB* allele, the mode(s) of inactivation of PDGF-Rβ encoded by the *PDGFRB* mutations remains unclear. Since the mutant PDGF-Rβ proteins are expressed, they may, in principle, work as dominant-negative proteins. Assuming that only PDGF-Rβ dimers consisting of two wild-type subunits have the capacity to signal, a dominant-negative mutant would reduce total signaling strength by a maximum of 75% [29]. We have previously reported that two transgenic *Pdgfb* hypomorphs: *Pdgfb* ^*ret/ret*^ and *Pdgfb* ^−/−^;*R26P* ^+/0^ mice form age-dependent pericapillary nodules that progressively get calcified [15]. While these mice constitute mouse models of PFBC, we suspected that the degree of PDGF-B signaling impairment in those models exceeds the one in human PFBC.

**Fig 7:**
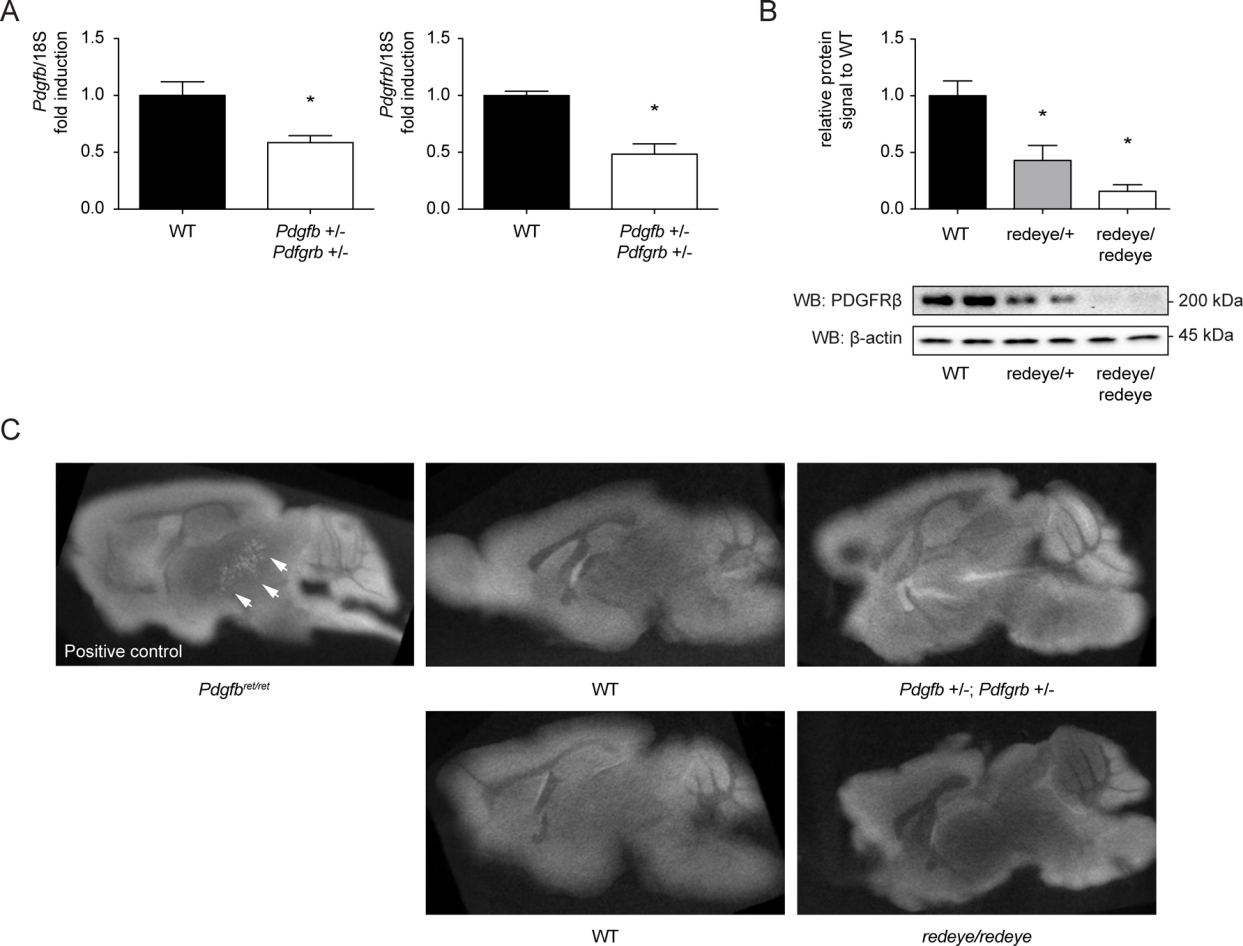
Screening for calcifications in *Pdgfb^+/-^; Pdgfrb^+/-^* and *Pdgfrb^redeye/redeye^* mice. (A). RT-PCR for murine *Pdgfb* and *Pdgfrb* in brain total RNA extractions. mRNA levels of *Pdgfb* and *Pdgfrb* are shown relative to the expression in wild-type animals. *p<0.05 as compared to wild-type animals. Error bars indicate standard deviation of mRNA levels in 4 different animals. (B). Western Blot for PDGFRβ levels in *Pdgfrb* ^redeye/redeye^ mice. A representative Western blot indicates the reduction in detectable protein level of PDGFRβ in two mice for each genotype. As a loading control, β-actin was used. The graphs display a quantification of the levels of PDGFRβ. Error bars indicate standard deviation of receptor levels from 4 animals each. *p<0.05 as compared to wild-type animals. (C). Micro computed tomography (micro-Ct) analysis of *Pdgfb* ^*ret/ret*^, *Pdgfb* ^*+/-*^;*Pdgfrb* ^*+/-*^ and *Pdgfr* ^*redeye/redeye*^ mouse brains. After perfusion, the mouse brain of aged mice was surgically removed and prepared for micro-Ct. For each genotype, three brains were scanned. Brains of *Pdgfb* ^*ret/ret*^ mice were used as a positive control, and *Pdgfb* ^*ret/+*^, *Pdgfrb* ^*redeye/+*^ or wild-type mice were used as negative controls.

In order to more closely mimic the degree of loss of PDGF-Rβ signaling deduced from our analysis of the mutant human PDGF-B and PDGF-Rβ proteins, we investigated other mouse models of *Pdgfb* and/or *Pdgfrb* hypomorphism. We first bred mice heterozygous null for *Pdgfb* and *Pdgfrb* (*Pdgfb* ^+/-^;*Pdgfrb* ^+/-^) and allowed them to age. This situation combines the features of the *PDGFB* haploinsufficiency observed with most *PDGFB* mutations with the decreased PDGF-Rβ expression seen in the R987W mutant. The combined loss of 50% of PDGF-B and 50% of PDGF-Rβ would also be predicted to lead to a 75% total reduction of PDGF-Rβ signaling strength, thereby mimicking a putative dominant-negative PDGF-Rβ mutant. Doubly heterozygous mice null for *Pdgfb* and *Pdgfrb* (*Pdgfb* ^+/-^;*Pdgfrb* ^+/-^) were viable and did not display any overt phenotype. The brain mRNA levels of *Pdgfb* and *Pdgfrb* were near half of those in wild-type, suggesting little or no compensation of the haploinsufficiency at the mRNA level (Fig 7A). Whereas *Pdgfb* ^ret/ret^ mice display calcified nodules as early as 4 months after birth, which progressed in size and number with age, as previously reported [15], *Pdgfb* ^+/-^;*Pdgfrb* ^+/-^ mice did not display any signs of brain calcification by Von Kossa staining (data not shown) or micro-CT brain scans (Fig 7C), even at advanced age (12–14 months) when such lesions are prevalent in *Pdgfb* ^ret/ret^ mice (Fig 7C).

To further lower the gene dose of *Pdgfrb*, we took benefit of the recently described *redeye* mouse mutant [30]. This mouse strain carries a *Pdgfrb* mutation at position +2 of intron 6, causing partial loss of normal splicing. Retaining intron 6 results in a frame shift and premature termination of the protein, which results in an estimated 75% reduction in the amount of *Pdgfrb* mRNA in homozygous mice [30]. *Pdgfrb* ^*redeye/redeye*^, *Pdgfrb* ^*redeye/+*^ and wild-type mice were bred and analyzed at 6 months. Western blot for PDGF-Rβ performed on brain lysates demonstrated an even more dramatic decrease in protein signal than reported [30], reaching only 10% of the wild-type levels in *Pdgfrb* ^*redeye/redeye*^ mice (Fig 7B). Similarly to *Pdgfb* ^+/-^;*Pdgfrb* ^+/-^ mice, we did not detect vessel-associated calcifications in *Pdgfrb* ^*redeye/redeye*^ mice using various histostains (data not shown) or by micro-CT scans (Fig 7C). Thus, in mice, haploinsuffiency for *Pdgfb* and *Pdgfrb*, as well as a substantial decrease in PDGF-Rβ protein levels caused by a splicing mutant, were insufficient to cause a PFBC-like pathology.

### The vascular calcification process does not correlate with pericyte loss and blood-brain barrier dysfunction

**Fig 8:**
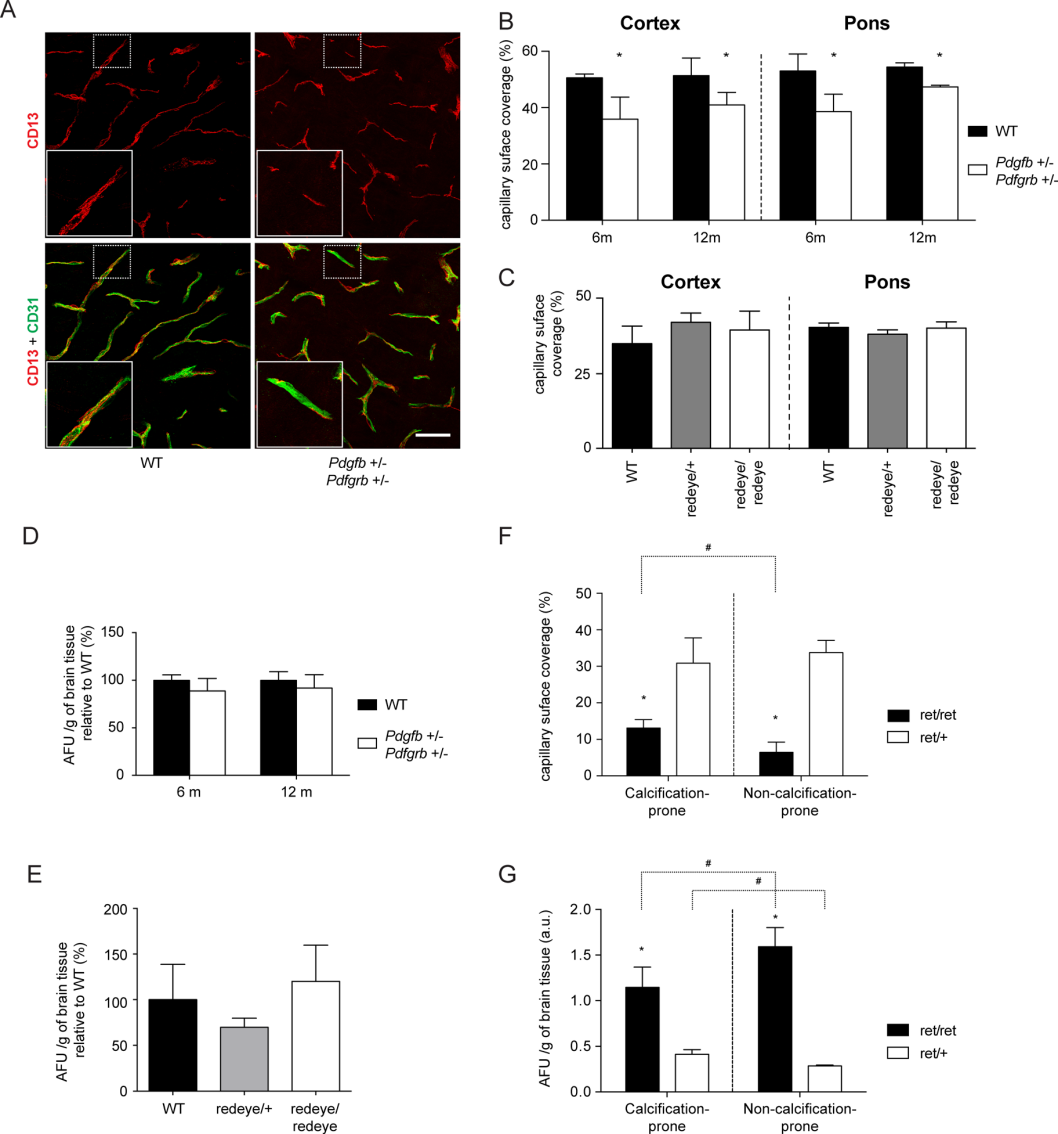
Assessment of vessel pericyte coverage and blood-brain barrier integrity in aged *Pdgfrb^redeye/redeye^* and *Pdgfb^+/-^; Pdgfrb^+/-^* mice. (ABC) Assessment of pericyte coverage. A CD13 and CD31 co-immunolabelling was performed on 50 μm-thick parasagittal vibratome sections. (A) Representative 2D projections of ~40 μm z-stacks taken from the dorsal pons of wild-type (left panels) and double heterozygous (right panels) animals; upper panels (red): CD13 immunostaining; lower panels: merged CD31 (green) and CD13 immunostainings. Inserts provide a more detailed indication of the coverage rate. Scale bar: 30 μm. The capillary surface coverage rate in *Pdgfb* ^*+/-*^ *; Pdgfrb* ^*+/-*^ (B) and *Pdgfrb* ^*redeye/redeye*^ mice (C) was calculated and is plotted as the percentage of the vessel surface enveloped by pericytes. Two pictures from the dorsal pons and two pictures from the cortex were analyzed for each animal. The standard deviation of pericyte coverage of 4 animals per genotype is indicated by the error bars. WT mice and heterozygous *Pdgfrb* ^*redeye/redeye*^ mice were used as controls. (DE) Blood-brain barrier permeability assessment in *Pdgfb* ^*+/-*^ *; Pdgfrb* ^*+/-*^ (D) and *Pdgfrb* ^redeye/redeye^ (E) mice. Lysine-fixable cadaverine conjugated to Alexa Fluor-555 was injected intravenously into the tail vein (5 mg/ml in saline) 2 hours before sacrifice. Fluorescence was measured in the brain homogenate and arbitrary fluorescence units (AFU) were normalized to the brain weight. Error bars indicate standard deviation of fluorescence level measurement in 4 different animals. (F). Pericyte coverage of vessels in calcification prone regions compared with non-calcification-prone regions. Different brain regions were assessed for pericyte coverage in *Pdgfb* ^ret/ret^ mice and the result is plotted as the percentage of the vessel surface enveloped by pericytes. For each animal, two pictures from the dorsal pons (calcification-prone) and two pictures from the cortex (non-calcification-prone) were analyzed. The standard deviation of pericyte coverage of 4 animals per genotype is indicated by the error bars. *p<0,05 when comparing *Pdgfb* ^ret/ret^ with *Pdgfb* ^ret/+^, #*p*<0,05 when comparing calcification-prone regions with non-calcification-prone regions in *Pdgfb* ^ret/ret^ mice. (G). Analysis of blood-brain barrier integrity in calcification prone regions compared to non-calcification-prone regions. Alexa Fluor-555 conjugated cadaverine tracer was allowed to circulate for 2 hours prior to sacrifice of the mice. The calcification prone regions of the brain were microdissected, and after homogenizing of the tissue, fluorescence was measured and normalized over the tissue weight (AFU). The standard deviation of 4 animals per genotype is indicated by the error bars. *p<0,05 when comparing *Pdgfb* ^ret/ret^ with *Pdgfb* ^ret/+^, #*p*<0,05 when comparing calcification-prone regions with non-calcification-prone regions in *Pdgfb* ^ret/ret^ mice.

Since our previous analysis established a correlation between brain calcifications and the loss of pericytes in *Pdgfb* ^*ret/ret*^ and *Pdgfb* ^−/−^;*R26P* ^+/0^ mice, we next assessed the pericyte coverage of the small vessels in *Pdgfb* ^+/-^;*Pdgfrb* ^+/-^ and *Pdgfrb* ^*redeye/redeye*^ mice, in search for age-dependent pre-calcification changes. *Pdgfb* ^+/-^;*Pdgfrb* ^+/-^ mice displayed a modest 20–30% decrease in pericyte coverage (Fig 8A and 8B), that either worsened with ageing (29±15% decrease at 6 months and 20±9% decrease at 12–14 months) nor varied according to anatomical location (13±1% decrease in the dorsal pons, a calcification prone region in *Pdgfb* ^*ret/ret*^ mice, as compared with 19±11% decrease in the cortex, a calcification-*non*-prone region in *Pdgfb* ^*ret/ret*^ mice) (Fig 8B). *Pdgfrb* ^*redeye/redeye*^, *Pdgfrb* ^*redeye/+*^ and wild-type mice did not display any significant decrease in pericyte coverage in the dorsal pons and in the cortex (Fig 8C).

As *Pdgfb* ^*ret/ret*^ and *Pdgfb* ^−/−^;*R26P* ^+/0^ mice also display an impaired blood-brain barrier the degree of which correlates with brain calcification, we investigated passage of exogenous tracer cadaverine-Alexa 555 from the blood into the brain. In *Pdgfb* ^+/-^;*Pdgfrb* ^+/-^ (Fig 8D), as well as in *Pdgfrb* ^*redeye/redeye*^ mice (Fig 8E), the blood-brain barrier permeability to the tracer was similar to controls.

*Pdgfb* ^*ret/ret*^ mice invariably develop calcifications in specific brain regions (thalamus, pons, mesencephalon, basal forebrain) from 4 months of age while displaying a general reduction in pericyte coverage of brain capillaries and increased permeability of the blood-brain barrier. In contrast, *Pdgfb* ^+/-^;*Pdgfrb* ^+/-^ mice presented only a minor decrease in vessel pericyte coverage and no vessel-associated calcification. These data strengthen the previously observed correlation [15] between the degree of overall brain pericyte loss and BBB impairment and the formation of brain calcification. However, certain brain regions (e.g. cerebral cortex) with severe pericyte loss and BBB deficiency did *not* develop brain calcifications. Therefore, we further investigated the spatial relationship between pericyte coverage, blood-brain barrier permeability and the formation of calcified nodules in *Pdgfb* ^*ret/ret*^ mice. Surprisingly, we found that the pericyte coverage was significantly higher in the 3 calcification-prone regions (thalamus, mesencephalon and dorsal pons; 13±3%) compared to 3 calcification-*non*-prone regions (motor cortex, hippocampus and myelencephalon; 6±2%) (Fig 8F and S3 Fig). Likewise, BBB permeability was increased 2 to 6-fold as compared with *Pdgfb* ^*ret/+*^ mice in all brain regions, but the vasculature in calcification-prone regions was significantly less permeable than in calcification-*non*-prone regions (Fig 8G and S3 Fig), mostly because of the high BBB permeability of telencephalic structures (cortex, hippocampus and striatum, S3 Fig). Hence, within *Pdgfb* ^*ret/ret*^ mice, the occurrence of vascular calcifications did not spatially correlate with the degree of pericyte loss and BBB leakage (see overview in S1 Table).

## Discussion

PFBC is an age-dependent neurological disease characterized by the formation of granular calcifications in specific regions of brain parenchyma [16]. Since the first autopsy study in 1850 [31], strong evidence have accumulated that PFBC is primarily a vascular disease. Located around the brain capillaries [32,33], most calcified nodules seem to form within the vascular basement membrane, possibly nucleating in the pericytes [34]. The identification of familial monogenic forms of PFBC resulting from mutations in *PDGFB* and *PDGFRB* strengthened the link between PFBC and vasculature. However, the extent to which human mutations alter PDGF-B signaling and impact pericyte recruitment, maintenance and function has remained unclear. In the present study, we demonstrate that the six *PDGFB* mutations analyzed are all *null* or loss-of-function alleles. We confirm that at least one of the *PDGFRB* mutation (L658P) results in a complete, or nearly complete, loss of signaling capacity [18,21]. We also demonstrate that, comparable degrees of genetic haploinsufficiency in mice did not cause brain calcification, and moreover, that a more pronounced (≈90%) decrease in PDGF-Rβ protein level also did not cause brain calcification. Somewhat surprisingly, these situations of limited PDGF-Rβ signaling did also not cause significant decreases in pericyte coverage or impairment of the BBB (see overview in S1 Table).

### *PDGFB* mutations

*PDGFB* encodes a PDGF-B protein that makes up a biologically active PDGF-BB dimer through disulfide bridging of cysteine residues 124 and 133 [35]. Heterozygous *PDGFB* mutations can impact PDGF-BB processing and function by abolishing protein synthesis or yielding an inactive protein that does not interfere with wild-type PDGF-B processing. This would lead to a theoretical 50% decrease in PDGF-BB through haploinsufficiency without compensatory mechanisms. Indeed, doubly heterozygous *Pdgfb* ^+/-^;*Pdgfrb* ^+/-^ mice lack compensatory upregulation of mRNA from the intact alleles (Fig 7A). The recently described intragenic deletion involving 4 out of 6 exons in *PDGFB* probably leads to a pure haploinsufficiency [20]. The missense mutation in the signal peptide (L9R) probably functions as a heterozygous null as well, because an N-terminal processing-deficient PDGF-B would fail to be translocated into the endoplasmic reticulum for further processing and secretion.

However, an inactive PDGF-B, which retains the ability to dimerize is potentially dominant-negative, and would be predicted to cause a 75% decrease in active PDGF-BB levels. The nonsense mutations (Q145*, Q147* and R149*), which are all situated downstream of the cysteine residues engaged in inter-chain bridging (Fig 1A), could potentially retain the ability to dimerize, as could the M1? and *242Yext*89 mutant proteins. While an intracellular protein product was only detectable for the two latter (Fig 2B), it remains possible that the former mutations do yield a protein product that is either immediately degraded or not recognized by the antibodies. Finally, the missense mutation in the receptor-binding loop (L119P) could hinder binding of the PDGF-B pro-peptide to the mature chain. The corresponding leucine in PDGF-A (L118) was shown to be essential for correct protein folding and secretion [36]. Placing the PFBC PDGF-B mutations on a ribbon model of PDGF-B indeed suggests that L119 (green in Fig 1B) is located on the outer surface of the L1 loop (grey in Fig 1B), among a large cluster of hydrophobic amino acids similar to L118 in PDGF-A chain.

Thus, Q145*, Q147*, R149* and L119P may all be unstable proteins that are rapidly targeted for intracellular degradation. In contrast the M1? and *242Yext*89 proteins accumulated intracellularly at levels that were comparable to wild-type PDGF-B, but with abnormal sizes corresponding to alternative translation initiation and C-terminal extension, respectively. A PDGF-Rβ binding-based ELISA assay detected only the *242Yext*89 product in conditioned cell culture medium, suggesting that if any of the other mutant proteins are secreted, they fail to accumulate to significant levels extracellularly. Finally, although the *242Yext*89 product bound to PDGFRβ epitopes in the ELISA assay, it failed to induce any detectable PDGF-Rβ autophosphorylation or any of the cellular cytoskeletal rearrangements typical for wild-type PDGF-BB. In conclusion, our analysis suggests that all PFBC mutations in *PDGFB* lead to loss of a functional PDGF-B protein.

### *PDGFRB* mutations

Regarding *PDGFRB* mutations, we confirm and complement recently reported data. In their recent study, Sanchez-Contreras *et al*. [18], transfected HeLa cells with different *PDGFRB*-expressing constructs, two carrying the L658P and R987W mutations [14], and two carrying two novel R695C and A1096V mutations identified in autopsy material stored in a brain bank of sporadic PFBC patients [18]. While the A1096V mutant behaved like the wild-type and was assumed to represent a benign variant, the L658P and R695C mutations resulted in defective PDGFRβ autophosphorylation. The R987W mutation led to decreased levels of protein [18]. We herein confirm the reported changes in L658P and R987W using two different cell types, HEK and PAE cells, the latter of which allowed us to assess also relevant cellular responses to PDGF-Rβ phosphorylation. *PDGFRB*-transfected PAE cells are a model of choice to study PDGF receptor function because they lack endogenous PDGF receptors, but contain all the necessary downstream effectors, and they respond similar to endogenously PDGF-Rβ-expressing fibroblasts by actin reorganization and chemotaxis upon PDGF-Rβ stimulation [26].

The L658P mutant lacked signaling ability, most likely because the new proline residue is close to the ATP-binding pocket in a highly conserved region (Fig 1C and S4 Fig) [14]. For the other two mutants, R987W and E1071V, our results are more ambiguous. We could reproduce the slight decrease in protein levels of the R987W mutant, as seen by Sanchez-Contreras *et al*., but the *in vivo* relevance of this decrease is less clear, as an animal model with extreme reduction in functional PDGF-Rβ expression (*Pdgfrb* ^*redeye/redeye*^) has no detectable vessel-associated calcifications (Fig 7). Alternatively, the R987W mutated receptor is disease-causing through its impaired signaling potential, as seen in the reduced activation of ERK1/2, AKT, and especially PLCγ (Fig 5). Indeed, we observed reduced phosphorylation levels of residue Y1009 in R987W (S2 Fig), which, together with Y1021, is necessary for PLCγ phosphorylation and activation [37]. PDGF-BB is a potent and selective stimulator of inorganic phosphate transport in vascular smooth muscle cells, an effect principally mediated by PLCγ [38]. This possible link between PDGF-Rβ, PLCγ and phosphate transport deserves further investigation, since mutations in the inorganic phosphate transporter Pit-2 (SLC20A2) [39], and the phosphate exporter XPR1 [40] are also linked to PFBC.

While R987W affects a phylogenetically conserved arginine residue in the C-terminal domain, the E1071V mutation is situated in the phylogenetically variable C-terminus of PDGF-Rβ (S4 Fig). In addition, its effect on downstream signaling is less pronounced than R987W, suggesting that this mutation might represent a benign polymorphism. Interestingly, although all three *PDGFRB* mutations displayed significantly less membrane ruffles when stimulated with PDGF-BB, this did not translate into impaired migration of PAE cells expressing R987W or E1071V PDGF-Rβ mutants as measured *in vitro* in a scratch “wound healing” assay (Fig 6). One possible explanation for this is that endothelial migration is regulated mostly though ERK1/2 and Akt activity [38], and to a lesser extend through PLCγ activity.

### How does impaired PDGF-B/ PDGF-Rβ signaling cause PFBC?

Our analysis establishes that mutations in *PDGFB* and *PDGFRB* associated with PFBC cause loss or reduced formation or function of the gene product. However, it remains unclear how impaired PDGF-B/PDGF-Rβ signaling causes brain calcification. The *PDGFB* and *PDGFRB* mutations described so far are dominant heterozygous mutations. Homozygosity for the same mutations may potentially cause pre- or perinatal lethality, similar to the situation in mouse homozygous knockouts [5,6]. In contrast to the situation in humans, however, heterozygous mouse knockouts of *Pdgfb* or *Pdgfrb* (not shown), and even the double heterozygotes *Pdgfb* ^+/-^;*Pdgfrb* ^+/-^ mice, did not develop brain calcifications (Fig 7). We reasoned that *Pdgfb* ^+/-^;*Pdgfrb* ^+/-^ should mimic the L658P mutation in the sense that both would be predicted to cause ≈75% loss of signaling, in *Pdgfb* ^+/-^;*Pdgfrb* ^+/-^ mice due to the combined 50% reduction of PDGF-B and PDGFRβ levels, and in the case of L658P, by an expected dominant-negative effect in receptor dimers. In a further attempt to mimic the L658P mutation in mice we investigated *Pdgfrb* ^*redeye/redeye*^ mice, which display a 75% reduction of *Pdgfrb* transcript levels [30] and a 90% reduction in PDGF-Rβ protein levels (Fig 7B). Intriguingly, also *Pdgfrb* ^*redeye/redeye*^ mice failed to develop brain calcifications.

Thus, our data suggest that humans and mice differ with regard to the threshold levels of PDGF-B/PDGF-Rβ signaling required to protect against brain calcification. Although PDGF-Rβ signaling levels have not been directly measured in *Pdgfb* ^*ret/ret*^ and *Pdgfb* ^−/−^;*R26P* ^+/0^ mice, which develop brain calcifications [15], we conclude that it is likely lower compared to *Pdgfrb* ^*redeye/redeye*^ and *Pdgfb* ^+/-^;*Pdgfrb* ^+/-^ mice. This conclusion is based on analysis of brain vessel coverage of pericytes, which is dependent on PDGF-B/PDGF-Rβ signaling. We previously reported a correlation between the degree of pericyte deficiency in brain microvessels, BBB impairment and the extent and onset age of brain calcification [15]. In our present analysis, we found that the pericyte coverage was 70–80% in *Pdgfb* ^+/-^;*Pdgfrb* ^+/-^ mice compared to wild-type mice (Fig 8B), a ratio comparable to the previously analyzed *Pdgfb* ^−/−^;*R26P* ^+/+^ mice (72% pericyte coverage compared to normal), which also lack brain calcifications [41]. Somewhat surprisingly, *Pdgfrb* ^*redeye/redeye*^ mice did not display any measurable reduction in pericyte coverage despite a 90% reduction of PDGF-Rβ protein levels. This may suggest that in mice, PDGF-B levels are more critically limiting for pericyte recruitment than PDGF-Rβ levels.

Taken together, these results show that in mice, more severely reduced levels of PDGF-B/PDGF-Rβ signaling are required in order to trigger brain calcifications than in humans. It is possible that the same holds true for pericyte and BBB deficiency. Histopathological signs of BBB defects have been reported in one PFBC case [33], but until now, pericyte densities have not been assessed in human PFBC cases. Thus, it remains unclear if pericyte loss and BBB defects correlate with brain calcification also in human PFBC.

While our results further strengthen the correlation between brain calcification, and defective PDGF-B/PDGF-Rβ signaling, pericyte loss and BBB impairment at a *global* level, we paradoxically did get opposite results when we analyzed the specific anatomical regions in *Pdgfb* ^*ret/ret*^ mice that develop brain calcifications in comparison with regions that do not. Here, we found that the calcification-prone regions had higher pericyte coverage and lower degree of BBB leakage than calcification-*non*-prone regions. Whereas these data do not rule out a role for pericyte loss and BBB impairment in brain calcification, it shows that these changes by themselves are not sufficient to cause microvascular calcification in any brain region, but that other region-specific factor(s) must also be involved. In this context it is interesting to note that the deep brain regions, such as the basal ganglia in humans, appear particularly sensitive to develop calcifications in conjunction with systemic mineral imbalance or aging in humans [16,42,43]. Current mouse models of PFBC also do not distinguish between the developmental role and a putative homeostatic function of PDGF-B signaling the brain vasculature. For example, it is possible that impaired PDGF-B/PDGF-Rβ signaling accelerates an age-dependent phenotypic change in pericytes that ultimately provokes the formation of microvascular calcification. Senescence and damage to vascular smooth muscle cells has been shown to cause vascular calcification of peripheral vessels [44]. These and other questions should be possible to address using inducible *Pdgfb* and *Pdgfrb* ablation as well as by crossing current models of PFBC, such as *Pdgfb* ^ret/ret^, into different sensitized or protective genetic backgrounds, and by exposing them to different systemic challenges, such as the induction of mineral imbalances.

## Material and Methods

### Generation of cDNA constructs carrying the PFBC mutations

Generation of cDNA constructs carrying the PFBC *PDGFB* mutations Human *PDGFB* transcript variant 1 cDNA (NM_002608.2) was bought from GeneCopoeia and subcloned into a pcDNA3.1 vector (Life Technologies). This construct contains the coding sequence for PDGF-B, as well as 255 bp upstream and 1105 bp downstream of the cDNA. To generate the L119P, Q145* and R149* *PDGFB* mutants, a QuickChange II Site-Directed Mutagenesis Kit (Agilent Technologies) was used. Primers were as follows: forward primer 5'-ACG CCA ACT TCC CGG TGT GGC CGC C-3' and reverse primer 5'-GGC GGC CAC ACC GGG AAG TTG GCG T-3' to generate L119P; forward primer 5'-GCC GCC CCA CCT AGG TGC AGC TG-3' and reverse primer 5'-CAG CTG CAC CTA GGT GGG GCG GC-3' to generate Q145*; forward primer 5'-CCA GGT GCA GCT GTG ACC TGT CCA GGT-3' and reverse primer 5'-ACC TGG ACA GGT CAC AGC TGC ACC TGG-3' to generate R149*. The remaining four mutations, namely M1?, L9R, Q147* and *242Yext*89, were generated in the original pcDNA3.1 PDGFB construct by GenScript (GenScript USA Inc). All constructs were verified by sequencing.

Constructs of human wild-type and kinase-dead (KD) *PDGFRB* cDNA subcloned into the pcDNA3.1 vector (Life Technologies) were described previously [26]. The KD construct bore the K634A mutation in which the nucleotide-binding lysine of the protein-tyrosine domain has been changed to alanine [26]. To generate the three *PDGFRB* mutants, namely L658P, R987W and E1071V, a QuikChange II Site-Directed Mutagenesis Kit was used. Primers were as follows: forward primer 5'-GAT CAT GAG TCA CCC TGG GCC CCA CCT GA-3' and reverse primer 5'-TCA GGT GGG GCC CAG GGT GAC TCA TGA TC-3' to generate L658P; forward primer 5'-CCA CCC AGC CAT CCT TTG GTC CCA GG-3' and reverse primer 5'-CCT GGG ACC AAA GGA TGG CTG GGT GG-3' to generate R987W; forward primer 5'-CAG GAC GAA CCA GTG CCA GAG CCC CAG-3' and reverse primer 5'-CTG GGG CTC TGG CAC TGG TTC GTC CTG-3' to generate E1071V. All constructs were verified by sequencing.

### Cell culture and transfection

Human embryonic kidney (HEK293) cells were purchased from ATCC and cultured in Dulbecco’s modified Eagle’s medium (DMEM) supplemented with 10% (v/v) fetal bovine serum, 2 mM L-glutamine, 10 U/ml penicillin, and 10 μg/ml streptomycin. Porcine Aortic Endothelial (PAE) cells [45] were cultured in DMEM and Ham’s F12 nutrient mixture (DMEM:F12) supplemented with 10% (v/v) fetal bovine serum, 2 mM L-glutamine, 10 U/ml penicillin, and 10 μg/ml streptomycin. Human brain vascular pericytes (HBPs) were purchased from ScienCell and cultured under standard culture conditions according to the manufacturer’s protocol.

cDNA vectors were introduced into the cells by Lipofectamine Plus transfection (Life Technologies). Cells were transfected at 70% confluency with 2.5 μg of DNA, 7 μl of Lipofectamine, 2.5 μl of Plus reagent and 200 μL OptiMEM (Life Technologies) per well in a 6-well plate. One day after transfection, the medium was removed and replaced by selection medium (normal growth medium with G418 sulfate 500 μg/ml, Life Technologies). To generate stable polyclonal colonies, cells were then kept in the selection medium and cultured at least three passages before use.

### RT-PCR and ELISA

Total RNA was extracted from HEK293 or PAE cells and DNase-treated using the RNeasy Mini kit (Qiagen). Total RNA was extracted for mouse brains and DNase-treated using the RNeasy Lipid Tissue Mini kit (Qiagen). cDNA was synthetized using the iScript™ cDNA Synthesis Kit (Bio-Rad). Real-time quantitative PCR was performed on a CFX96 Touch RT-PCR machine (Bio-Rad) using either SYBRgreen (iTaq Universal SYBR Green Supermix, Bio-Rad) or TaqMan probes (Life Technologies). In all conditions, eukaryotic 18S rRNA (4319413E) was used as a reference gene for ΔΔCt calculations. Since HEK293 cells express detectable levels of human PDGFB mRNA, their expression level was taken as 1 and the other values are expressed as fold increase over basal expression. When using SYBRgreen, the following primers were used. For detection of human *PDGFB*: forward primer 5’-GAT GAT CTC CAA CGC CTG-3’ and reverse primer 5’-TCC TTC TTC CAC GAG CCA-3’. For detection of human *PDGFRB*: forward primer 5’-ACC TGC AAT GTG ACG GAG GAG-3’ and reverse primer 5’-AAC ACT ACC TGC AGT GTC CG-3’. The following TaqMan probes were used: Mouse *Pdgfrb* (Mm00435546_m1) and mouse *Pdgfb* (Mm00440677_m1). All RT-PCR data is represented as the mean of three independent experiments.

The Quantikine ELISA Kit (R&D Systems) was used to measure the concentration of PDGF-BB in the conditioned media.

### PDGF-BB stimulation and Western blotting

Conditioned medium was prepared from serum-starved HEK293 cell transfected either with an empty-vector or with vectors encoding wild-type or mutant *PDGFB*. After 24 hours medium was collected, centrifuged and the supernatant was stored at -80°C until further use. Recombinant human PDGF-BB was purchased from R&D Systems. For PDGFRβ autophosphorylation assays, cells were starved in serum-free medium overnight and cooled on ice for 15 min before addition of 40 ng/ml PDGF-BB, conditioned medium collected from HEK cells transfected with plasmids encoding wild-type or mutant PDGF-Bs, or PDGF-BB dilution buffer only. Cells were kept on ice for 1 hour before lysis. For time-course analysis, PDGF-BB was directly added to the normal growth medium at 37°C to reach a concentration of 40 ng/ml. Cells were rinsed with ice-cold PBS and snap-frozen at different time-points. All cells were lysed in RIPA buffer (25 mM Tris-HCl pH 7.6, 150mM NaCl, 1% NP-40, 1% sodium deoxycholate, 0.1% SDS) containing 2 mM orthovanadate and protease inhibitors (Complete; Roche Molecular Biochemicals).

Unless stated otherwise, equal protein amounts from whole-cell extract were subjected to SDS/PAGE electrophoresis and electrotransferred to a PVDF membrane (Bio-Rad). Protein concentrations were determined using the BCA method (Thermo Scientific, Pierce). Proteins were visualized by incubating membranes with specific antibodies: rabbit monoclonal anti-PDGF-Rβ (28E1, Cell Signaling Technology); rabbit monoclonal anti-phospho-PDGF-Rβ (Y751), phospho-PDGF-Rβ (Y771), phospho-PDGF-Rβ (Y1009) and phospho-PDGF-Rβ (Tyr 1021) (Cell Signaling Technologies); rabbit monoclonal anti-phospho-p44/42 MAPK (ERK1/2) (Thr202/Tyr204) (Cell Signaling Technologies); rabbit polyclonal anti-phospho-Akt (Ser473) (Cell Signaling Technologies); rabbit anti-phospho-PLCγ1 (Tyr783) (Cell Signaling Technologies); rabbit anti-PDGF-BB antibody (AbCam). After incubation with an HRP-coupled secondary antibody, the membrane was soaked in a chemiluminescent substrate (Clarity Western ECL substrate, Bio-Rad). A high-resolution CCD camera (ChemiDoc MP, Bio-Rad) was used for signal detection and optimal exposure was determined by the software. Band intensities were analyzed by densitometry using Image Lab software (Bio-Rad).

For the autophosphorylation study, the amount of PDGF-Rβ was first determined in cell lysates by Western blotting. Lysates were then diluted to yield a comparable amount of PDGF-Rβ signal in each condition. The ratio of phospho/total PDGF-Rβ signal was calculated for each analyzed tyrosine residue and expressed as a percentage of the ‘wild-type PDGF-Rβ, PDGF-BB-treated’ condition, which was arbitrarily set to 100% in each individual experiment. Deviation from this value was analyzed with one-sample *t*-tests (mean = 100; two-tailed). For the downstream signaling study, the ratio to β-actin signal was calculated for each pathway and expressed as a percentage of the condition in which wild-type PDGF-Rβ was treated with PDGF-BB for 5 minutes, which was arbitrarily set to 100% in each individual experiment. Deviation from this value at 5 minutes was analyzed with one-sample *t*-tests (mean = 100; two-tailed). Deviation from wild-type PDGF-Rβ at other time points was analyzed using one-way ANOVAs followed by Dunnett’s multiple comparison tests.

Western blotting on mouse brain lysates was performed using the same method. One brain hemisphere per mouse was used for protein extraction. Homogenization was performed in RIPA buffer completed with protease and phosphatase inhibitors. Protein concentrations were determined in the supernatant using a BCA assay and normalized by dilution in RIPA. Ten μg total protein were loaded in each well.

### PDGF-BB-induced actin reorganization and *in vitro* scratch wound assays

To assess PDGF-BB-induced actin reorganization in PAE cells and HBPs, 5 x 10^4^ cells were seeded in glass-bottom 24 well-plates and cultured overnight. Subsequently, the cells were serum-starved overnight, cooled on ice for 15 min, and medium was replaced by either conditioned medium or fresh medium containing PDGF-BB. After a 30-min incubation on ice, cells were transferred back to the 37°C incubator for 15 min, then washed in phosphate-buffered saline (PBS) and fixed in 3.75% formaldehyde/PBS. Phalloidin staining was performed by incubating the cells in 3% bovine serum albumin (BSA) - 0.5% Triton X-100 /PBS for 1 hour, then in 2.5% PromoFluor 488-phalloidin (PromoKine) - 0.01% Hoechst 33342 (Life Technologies) - 1% BSA/PBS for 30 min. Cells were post-fixed for 2 min in 3.75% formaldehyde before mounting. Pictures were taken with a Leica TCS SP8 confocal microscope (Leica Microsystems) using a 63x objective (N/A 1.40). For quantification of membrane ruffles in PAE cells, 1 stitched image consisting of 9 adjacent images (3x3) was taken per condition (at 20x magnification, N/A 0.8). Ruffles were manually counted and the total ruffle count was normalized over the total amount of cells in the image. The latter were counted automatically using Fiji [46]. All images were deconvolved prior to analysis and presentation (AutoQuant X3, MediaCybernetics).

*In vitro* scratch wound assays were performed on stably transfected PAE cells grown until confluence in 96-well ImageLock Microplates (Essen Bioscience). After a 2 hour-starvation in serum-free medium, a single wound was made down the center of each well using the 96-well WoundMaker (Essen Bioscience). Cells were washed in PBS and then stimulated with 40 ng/ml PDGF-BB or vehicle. Wound images were then automatically acquired and registered -by the IncuCyte Zoom live-cell imager (Essen Bioscience) for every 2h until the wound was healed. Data were then analyzed in relative wound density/time. Relative wound density measures the spatial cell density in the wound area relative to the spatial cell density outside of the wound area at every time point. It is designed to be 0% at t = 0, and 100% when the cell density inside the wound is the same as the cell density outside the initial wound. This metric is calculated by custom algorithms, which are part of the IncuCyte software package (Essen BioScience).

### Animals and animal experiments

*Pdgfb* and *Pdgfrb* knockout mice [5,6] were bred to generate double heterozygous mice (*Pdgfb* ^+/-^;*Pdgfrb* ^+/-^). *Pdgfrb* ^redeye/+^ mice were obtained from the Medical Research Council (MRC), UK. Mouse Contract Services were provided by the Marie Lyon Centre at MRC Harwell (www.har.mrc.ac.uk). *Pdgfb* ^*ret/ret*^ mice have been described previously [41,47]. This study was carried out in strict accordance to the recommendations of the Swedish Ethical Committee on Animal Research. The protocol was approved by the Stockholm North Ethical Committee on Animal Research (Permit number N16/12) and by the Uppsala Ethical Committee on Animal Research (Permit number: C224/12 and C225/12). All surgery was performed under terminal anesthesia, and all efforts were made to minimize suffering.

For blood-brain barrier integrity assessment, lysine-fixable cadaverine (25 μg/g body weight) conjugated to Alexa Fluor-555 (Life Technologies) was injected intravenously into the tail vein 2 hours before sacrifice. Anaesthetized animals were perfused transcardially for 5 min with Hanks’ balanced salt solution (HBSS). Half of the forebrain and one kidney were removed and homogenized in 1% Triton X-100 /PBS, pH 7.2. Brain and kidney lysates were centrifuged at 14,000 rpm for 20 min and the relative fluorescence of the supernatant was measured using a Synergy HT microplate reader (BioTek) with the following settings: excitation: 540/25 nm, emission: 590/20 nm. To assess regional differences in the blood-brain barrier permeability, different brain regions were dissected under a stereomicroscope and weighed prior to homogenization, then processed the same way as half-brains. Kidneys were used as a control to ensure the systemic distribution of the injected tracers. Results were expressed as arbitrary fluorescent units normalized to the tissue weight (AFU/g).

For immunohistochemical studies, fifty μm sagittal vibratome sections of the mouse brains were successively incubated in blocking/permeabilization solution (1% bovine serum albumin, 0.5% TritonX-100 in PBS), primary antibody solution, and secondary antibody solution for 20 hours at 4°C. Sections were mounted in Prolong Gold antifade reagent (Life Technologies). The following primary antibodies were used: goat anti-mouse CD31 (R&D Systems); rat anti-mouse CD13 (AbD Serotec). Specimens were analyzed using a Leica TCS SP8 confocal microscope (Leica Microsystems). All immunohistochemistry images presented are 2D maximum intensity projections of ~40 μm z-stacks.

Pericyte coverage was estimated using a self-designed Fiji macro [46]. The estimation of capillary ensheathment by pericytes was performed by quantifying the ratio of CD31-positive area (in green) overlapped by CD13 (in red) in maximal intensity projections of the Z-stack. After transforming each layer into binary images using Tsai’s AutoThreshold function [48], layers were merged back and the ratio of yellow (colocalized) over yellow and green (CD31) pixels was calculated for each image. In each experiment, 4 animals per group were studied, and 2 images were acquired per animal for each region. Pericyte-coverage was determined in cortex and dorsal pons in *Pdgfb* ^+/-^;*Pdgfrb* ^+/-^ and *Pdgfrb* ^*redeye/redeye*^ mice and littermate controls, and in cortex, hippocampus, myelencephalon, thalamus, mesencephalon and dorsal pons in *Pdgfb* ^*ret/ret*^ mice and littermate controls.

Micro-CT scanning and histochemistry methods have been described previously [15]. In short, paraformaldehyde fixed whole-brain samples were incubated in iopromide (Ultravist 300, Bayer) to increase soft-tissue contrast. High-resolution data sets were obtained with a Skyscan 1176 small animal CT imager applying 50 kV tube voltage, 500mA tube current, an isotropic voxel size of 18 μm, and a 0.5mm aluminium filter. Image reconstruction was performed with a modified Feldkamp algorithm.

### Statistical analysis

Unless stated otherwise, statistical significance was determined by using unpaired two-tailed Student’s t-test For all statistical tests, p<0.05 was deemed significant. In graphs, error bars represent the SD from independent experiments; *, ^#^: p<0.05.

## Supporting Information

S1 Fig Conditioned medium from mutant *PDGFB*-transfected HEK cells fails to induce membrane ruffles in human brain pericytes. Overview demonstrating that the supernatant of mutant PDGF-B producing HEK293 cells does not induce circular ruffles in HBP cells. In addition, additional positive controls with exogenous PDGF-BB (40 ng and 100 ng per ml) are shown. Cyan: DAPI. Green: Alexa 488 conjugated phalloidin. Scale bar: 30 μm. Arrowheads point at representative ruffles.
(TIF)

S2 Fig Quantification of PDGF-Rβ autophosphorylation signal from tyrosine residues 751, 771, 1009 and 1021 using phospho-specific antibodies. Signals were normalized over total levels of PDGF-Rβ protein, then expressed as a percentage of wild-type PDGF-Rβ autophosphorylation. Shown in the graphs are the results from the 4 tyrosine residues that were assessed in three independent experiments. *p<0,05 when compared to the positive control (wild-type PDGF-Rβ).
(TIF)

S3 Fig Comparison of pericyte coverage and blood-brain barrier integrity between calcification-prone and non-calcification-prone regions in *pdgfb* ^*ret/ret*^ mice. (A) Pericyte coverage in different brain regions in *pdgfb* ^*ret/ret*^ mice. The data is plotted as the percentage of the vessel surface enveloped by pericytes. For each brain region, four pictures were analyzed. The standard deviation of pericyte coverage of 4 animals per genotype is indicated by the error bars. *p<0,05 when comparing *Pdgfb* ^ret/ret^ regions with the corresponding *Pdgfb* ^ret/+^ regions. (B) Blood-brain barrier integrity in different brain regions in *pdgfb* ^*ret/ret*^ mice. Fluorescent tracer was allowed to circulate for 2 hours prior to sacrifice of the mice. The different brain regions were micro-dissected, and after homogenizing of the tissue, fluorescence was measured and normalized over the tissue weight. The 3 calcification prone regions are grouped on the left of the graph. *p<0,05 when comparing *Pdgfb* ^ret/ret^ regions with the corresponding *Pdgfb* ^ret/+^ regions.
(TIF)

S4 Fig Amino acid sequence alignment of *PDGFRB* between orthologues. The human sequence of PDGF-Rβ was aligned to *Macaca fascicularis* (rhesus monkey), *Rattus norvegicus* (common rat), *Mus musculus* (house mouse) and *Gallus gallus* (red junglefowl). While L658P and R987W are highly conserved, E1071 is only conserved between *Homo sapiens* and *Macaca fascicularis*.
(TIF)

S1 Movie Time lapse imaging of wound closure of PAE cells expressing wild-type PDGF-Rβ and PDGF-Rβ L658P. The cells were imaged every hour for 13 hours to record wound healing. While wild-type PDGF-Rβ expressing cells were able to close the wound in the allotted time frame, the PDGF-Rβ L658P expressing cells failed to do so.
(MP4)

S1 Table Overview of the findings. (DOCX)
