# Supplementary figures and images for: Functional Characterization of Germline Mutations in PDGFB and PDGFRB in Primary Familial Brain Calcification
Source: PLoS One. 2015 Nov 23;10(11):e0143407. doi: 10.1371/journal.pone.0143407 (PMC4658112; doi:10.1371/journal.pone.0143407)

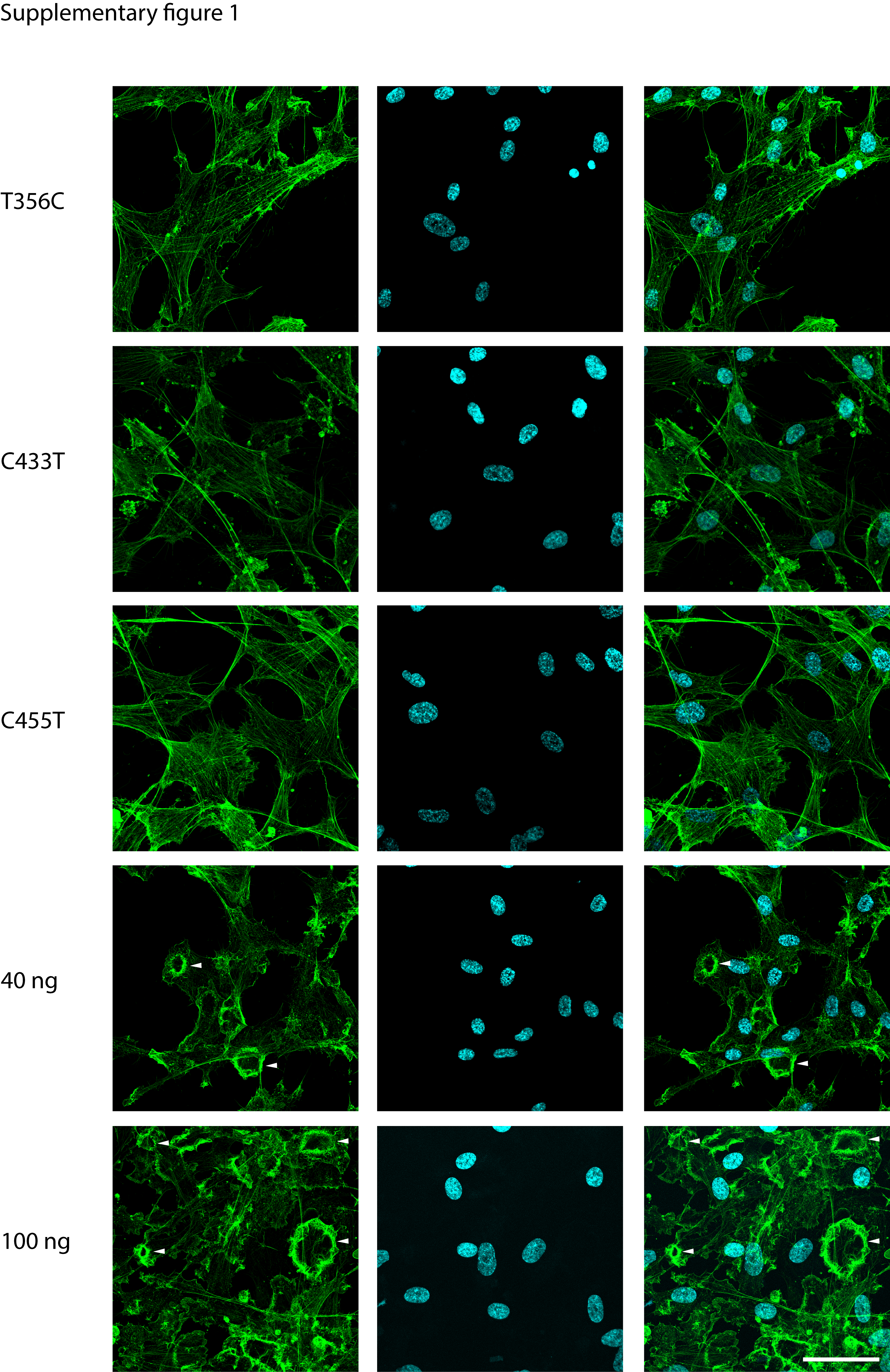

Supplement: S1 Fig — Overview demonstrating that the supernatant of mutant PDGF-B producing HEK293 cells does not induce circular ruffles in HBP cells. In addition, additional positive controls with exogenous PDGF-BB (40 ng and 100 ng per ml) are shown. Cyan: DAPI. Green: Alexa 488 conjugated phalloidin. Scale bar: 30 μm. Arrowheads point at representative ruffles. (TIF) [file pone.0143407.s001.tif]

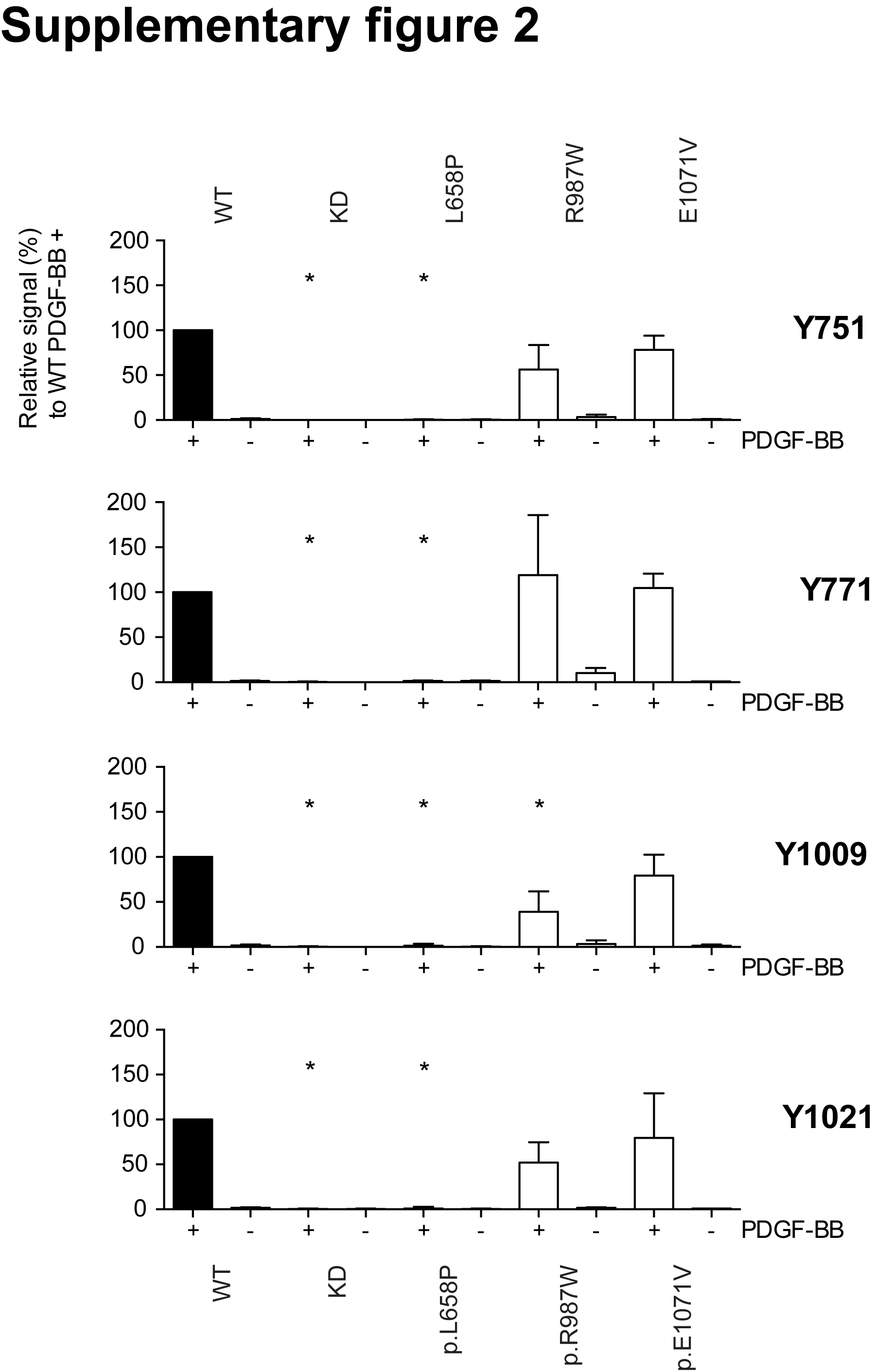

Supplement: S2 Fig — Signals were normalized over total levels of PDGF-Rβ protein, then expressed as a percentage of wild-type PDGF-Rβ autophosphorylation. Shown in the graphs are the results from the 4 tyrosine residues that were assessed in three independent experiments. *p<0,05 when compared to the positive control (wild-type PDGF-Rβ). (TIF) [file pone.0143407.s002.tif]

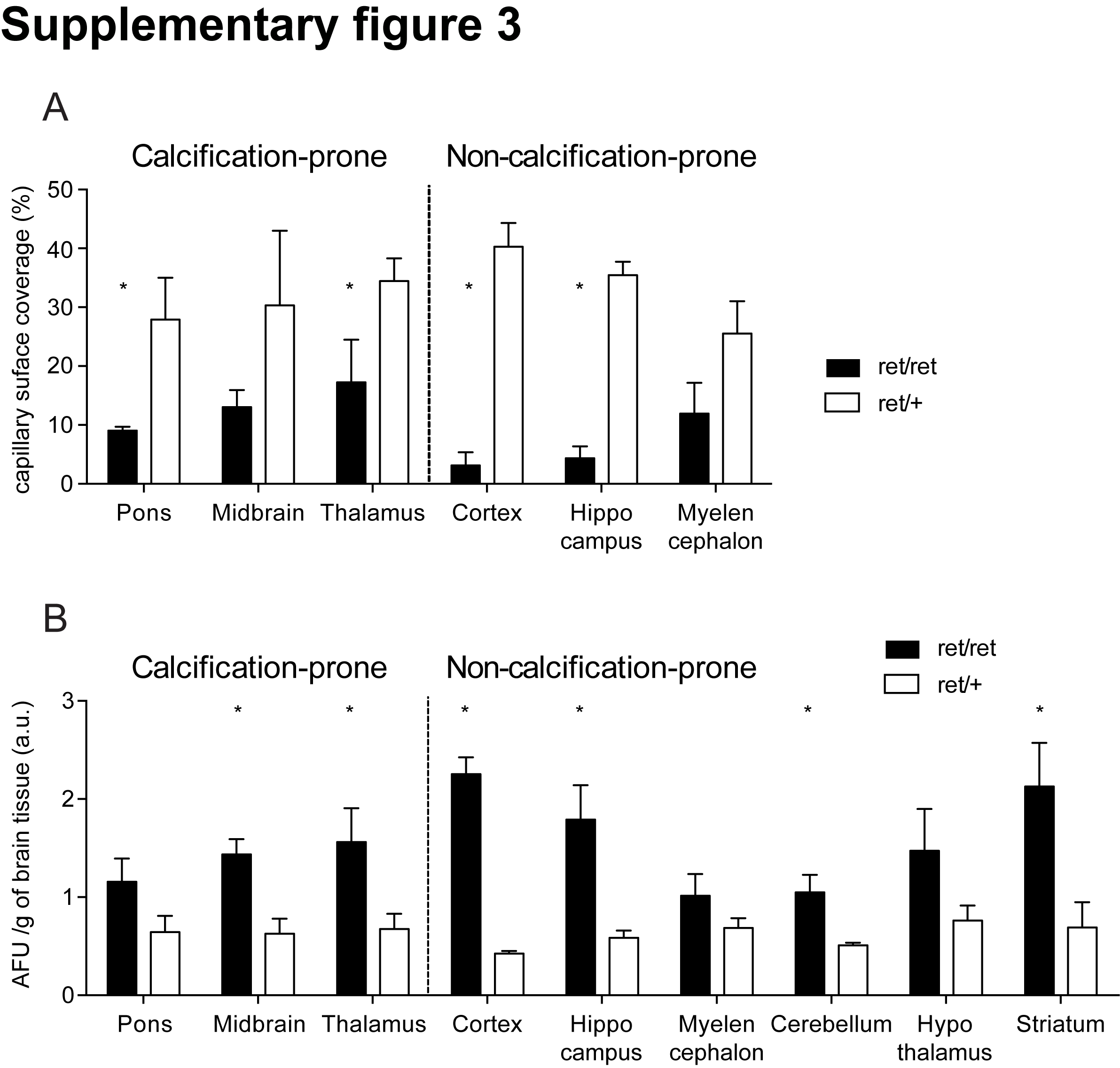

Supplement: S3 Fig — (A) Pericyte coverage in different brain regions in pdgfb ret/ret mice. The data is plotted as the percentage of the vessel surface enveloped by pericytes. For each brain region, four pictures were analyzed. The standard deviation of pericyte coverage of 4 animals per genotype is indicated by the error bars. *p<0,05 when comparing Pdgfb ret/ret regions with the corresponding Pdgfb ret/+ regions. (B) Blood-brain barrier integrity in different brain regions in pdgfb ret/ret mice. Fluorescent tracer was allowed to circulate for 2 hours prior to sacrifice of the mice. The different brain regions were micro-dissected, and after homogenizing of the tissue, fluorescence was measured and normalized over the tissue weight. The 3 calcification prone regions are grouped on the left of the graph. *p<0,05 when comparing Pdgfb ret/ret regions with the corresponding Pdgfb ret/+ regions. (TIF) [file pone.0143407.s003.tif]

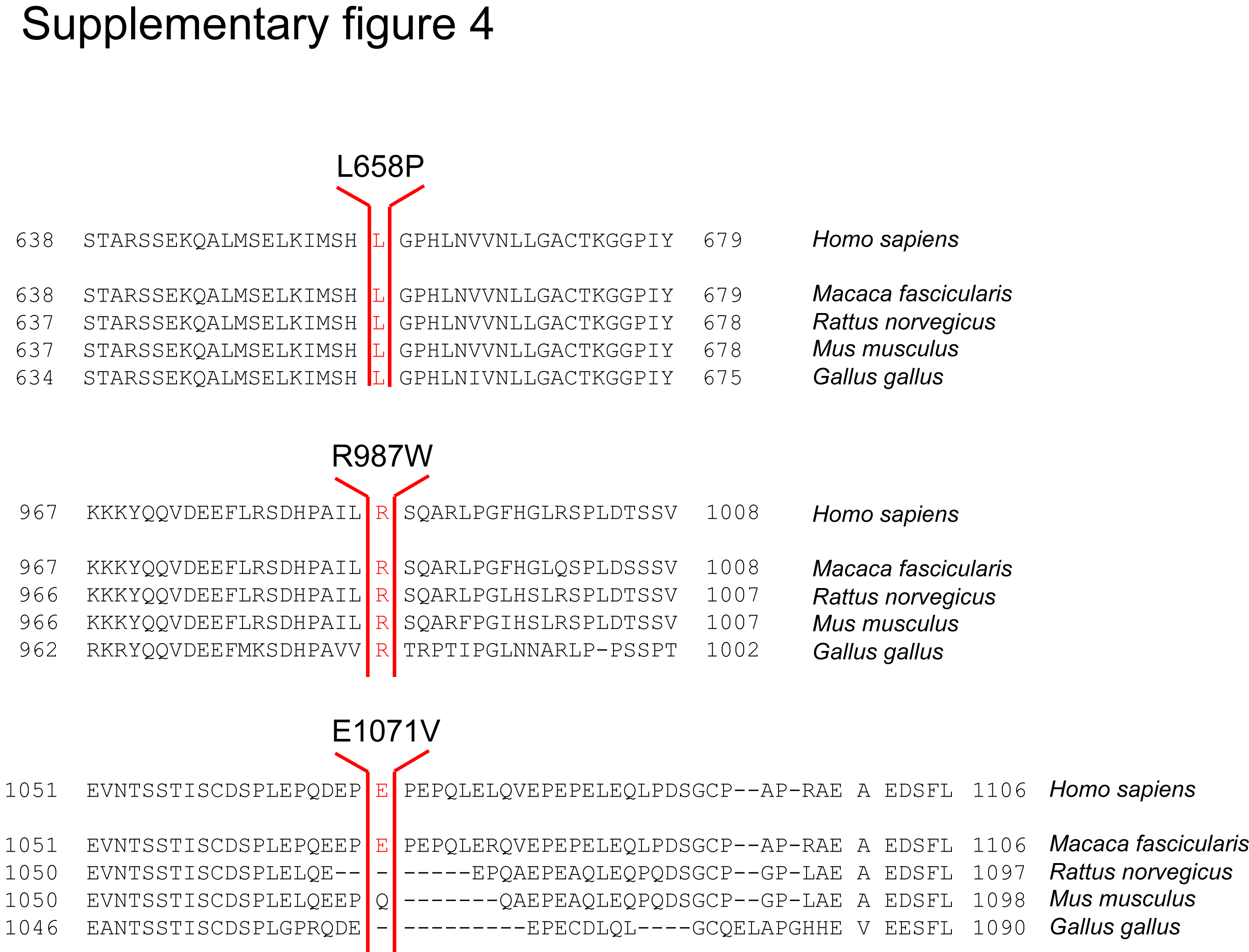

Supplement: S4 Fig — The human sequence of PDGF-Rβ was aligned to Macaca fascicularis (rhesus monkey), Rattus norvegicus (common rat), Mus musculus (house mouse) and Gallus gallus (red junglefowl). While L658P and R987W are highly conserved, E1071 is only conserved between Homo sapiens and Macaca fascicularis. (TIF) [file pone.0143407.s004.tif]
