# Supplementary material for: Functional Characterization of Germline Mutations in PDGFB and PDGFRB in Primary Familial Brain Calcification
Source: PLoS One. 2015 Nov 23;10(11):e0143407. doi: 10.1371/journal.pone.0143407 (PMC4658112; doi:10.1371/journal.pone.0143407)
Supplement: S1 Table — (DOCX) [file pone.0143407.s006.docx]

## Supplementary table 1. Overview of the findings

| ***In vitro* characterization of human *PDGFB* mutations** | | | | | | | | | | | | | | |
| --- | --- | --- | --- | --- | --- | --- | --- | --- | --- | --- | --- | --- | --- | --- |
| **PDGFB *mutation*** | | *Mutation* | | | *Detectable protein product by Western blot / ELISA* | | | | *Ability to phosphorylate PDGF-Rβ in pericytes* | | | *Ability to trigger ruffle formation in pericytes* | | |
| **M1?** | | Familial | | | **+**/- | | | | - | | | - | | |
| **L9R** | | Familial | | | -/- | | | | - | | | - | | |
| **L119P** | | Familial | | | -/- | | | | - | | | - | | |
| **Q145*** | | Familial | | | -/- | | | | - | | | - | | |
| **R149*** | | Familial | | | -/- | | | | - | | | - | | |
| **Gln147*** | | Sporadic | | | -/- | | | | - | | | - | | |
| **242Yext*89** | | Familial | | | **+/+** | | | | - | | | - | | |
| **In vitro characterization of human *PDGFRB* mutations** | | | | | | | | | | | | | | |
| **PDGFRB *mutation*** | *Mutation* | | | *Protein expression and stability* | | *Receptor autophos-phorylation* | | *Impaired downstream pathway* | | | *Ruffle formation* | | *Cell migration* | |
| **L658P** | Familial | | | Slightly decreased | | Impaired | | All (kinase dead) | | | Abolished | | Abolished | |
| **R987W** | Sporadic | | | Dramatically decreased | | Normal | | Akt and PLCγ? | | | Slightly decreased | | Normal | |
| **E1071V** | Sporadic | | | Normal | | Normal | | PLCγ? | | | Slightly decreased | | Normal | |
| **Mouse models with genetic *pdgfb* and/or *pdgfrb* insufficiency** | | | | | | | | | | | | | | |
| **Mouse model** | | | *Genetic insufficiency* | | | | *Peri-capillary calcifications* | | | *Pericyte coverage* | | | | *BBB leakage* |
| ***Pdgfb^+/-^; Pdgfrb^+/-^*** | | | 50% decrease in *pdgfb* and *pdgfrb* transcripts | | | | Absent | | | 20-30% decrease | | | | Absent |
| ***Pdgfrb^redeye/redeye^*** | | | 75% decrease in *pdgfrb* transcript  90% decrease in PDGF-Rβ protein | | | | Absent | | | Normal | | | | Absent |
| ***Pdgfb^ret/ret^*** | | | Severe reduction in perivascular PDGF-B concentration | | | | From 4 months | | | 65-85% decrease | | | | Present |
